# The *Esg* Gene Is Involved in Nicotine Sensitivity in *Drosophila melanogaster*

**DOI:** 10.1371/journal.pone.0133956

**Published:** 2015-07-29

**Authors:** Iván Sanchez-Díaz, Fernando Rosales-Bravo, José Luis Reyes-Taboada, Alejandra A Covarrubias, Verónica Narvaez-Padilla, Enrique Reynaud

**Affiliations:** 1 Departamento de Genética del Desarrollo y Fisiología Molecular, Instituto de Biotecnología, Universidad Nacional Autónoma de México, Avenida Universidad, 2001, Apartado Postal, 510–3, Cuernavaca 62210, México; 2 Centro de Investigación en Dinámica Celular, Universidad Autónoma del Estado de Morelos, Av. Universidad 1001, Col. Chamilpa, Cuernavaca, Morelos 62209, México; 3 Departamento de Biología Molecular de Plantas, Instituto de Biotecnología, Universidad Nacional Autónoma de México, Avenida Universidad, 2001, Apartado Postal, 510–3, Cuernavaca 62210, Mexico; University of Houston, UNITED STATES

## Abstract

In humans, there is a strong correlation between sensitivity to substances of abuse and addiction risk. This differential tolerance to drugs has a strong genetic component. The identification of human genetic factors that alter drug tolerance has been a difficult task. For this reason and taking advantage of the fact that *Drosophila* responds similarly to humans to many drugs, and that genetically it has a high degree of homology (sharing at least 70% of genes known to be involved in human genetic diseases), we looked for genes in *Drosophila* that altered their nicotine sensitivity. We developed an instantaneous nicotine vaporization technique that exposed flies in a reproducible way. The amount of nicotine sufficient to “knock out” half of control flies for 30 minutes was determined and this parameter was defined as Half Recovery Time (HRT). Two fly lines, *L4* and *L70*, whose HRT was significantly longer than control´s were identified. The L4 insertion is a loss of function allele of the transcriptional factor *escargot* (*esg*), whereas *L70* insertion causes miss-expression of the microRNA cluster *miR-310-311-312-313* (*miR-310^c^*). In this work, we demonstrate that *esg* loss of function induces nicotine sensitivity possibly by altering development of sensory organs and neurons in the medial section of the thoracoabdominal ganglion. The ectopic expression of the *miR-310^c^* also induces nicotine sensitivity by lowering Esg levels thus disrupting sensory organs and possibly to the modulation of other *miR-310^c^* targets.

## Introduction

Nicotine addiction is a serious public health problem. Several studies in humans have provided evidence that there is a strong genetic component underlying nicotine and other substances addiction.

Assessing the genetic contribution to addiction in humans is, to say the least, complicated. It is very difficult to separate the environmental contribution from the genetic effects; therefore, the use of animal models to study the genetic basis of addiction is invaluable. *Drosophila melanogaster* and humans respond to pharmacological treatments in very similar ways. Flies become hyperactive after the exposure to cocaine, fall unconscious after ingesting a sufficient dose of benzodiazepines and have a very similar intoxication with alcohol as humans do, thus they have consistently been used to study the genetic basis of drug differential sensitivity and addiction [1,2]. Considering that, in humans, hypersensitivity to drugs correlates with an increased addiction risk [3,4], we established a protocol for the reproducible exposure of flies to nicotine and characterized the normal response of wild type (wt) flies to this exposure. Using this approach, we identified two P-element insertion lines (*L4* and *L70*) that confer hypersensitivity to nicotine. *L4* insertion causes a loss-of-function mutation of the *escargot* (*esg*) gene, whereas *L70* insertion leads to the miss-expression of the *miR-310* ^*c*^. The *miR-310* ^*c*^ codes for four microRNAs: miR-310, miR-311, miR-312 and miR-313 that share an identical seed sequence. Furthermore, we show that ectopic expression of the *miR-310* ^*c*^ enhances *esg* phenotypes in the same way as the knock down of Esg by an RNAi, suggesting that these miRNAs downregulate *esg* expression. We also demonstrate that other *esg* loss-of-function alleles have a similar nicotine hypersensitive phenotype. It is important to note that this is the first time a behavioral phenotype is associated with the *miR-310* ^*c*^.

## Results

### *L4* and *L70 P{GawB}* insertions confer an abnormal sensitivity to nicotine

**Fig 1:**
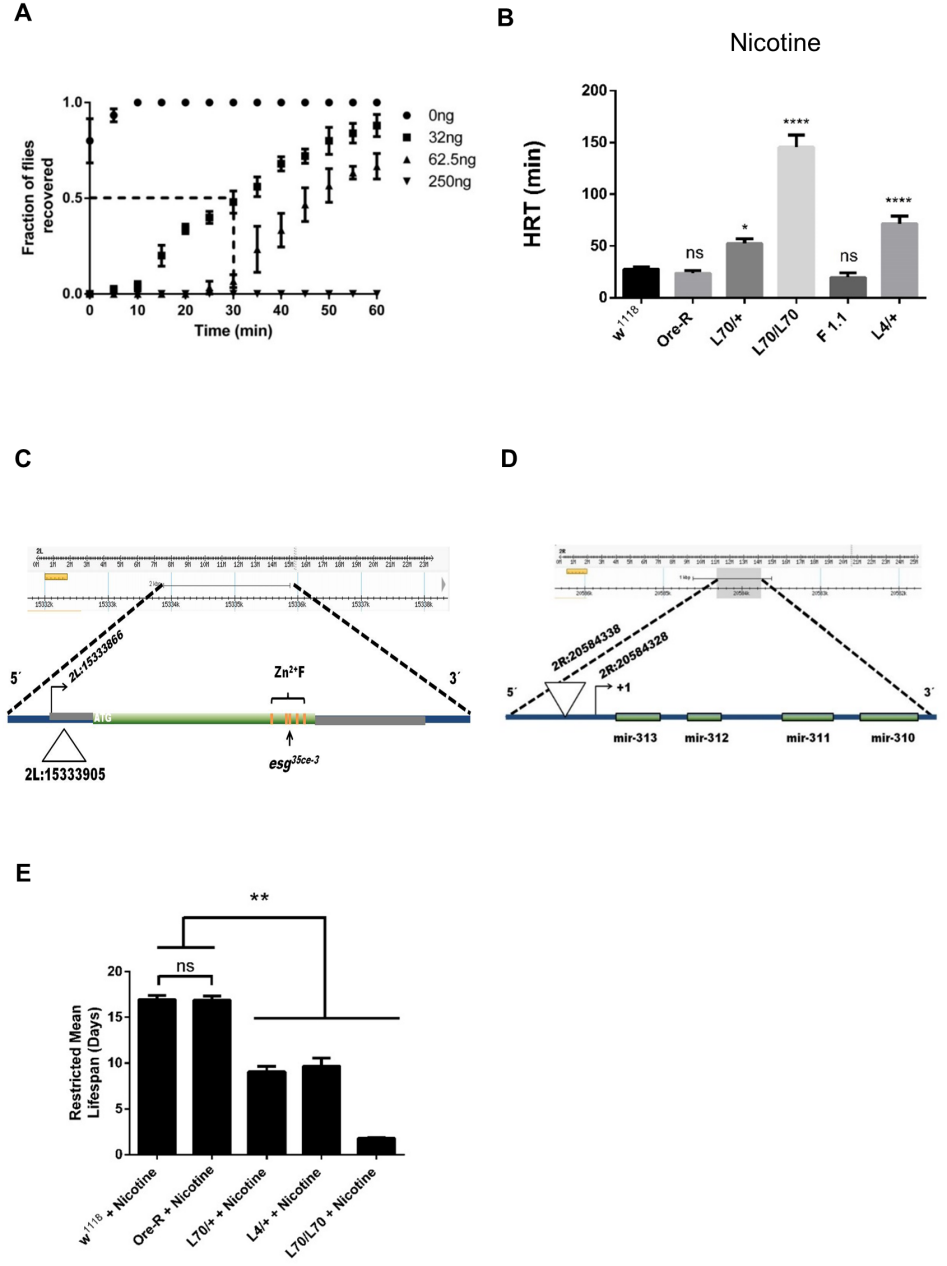
Genetic background determines *Drosophila* response to nicotine. (A) Dose response of *w* ^*1118*^ flies to different concentrations of nicotine. The time needed for half of the flies to recover from a given concentration of nicotine exposure is defined as Half Recovery Time (HRT), note that mock exposure of flies (0 ng, closed circles) allows flies to recover nearly immediately. (B) HRTs are shown for different fly genotypes: *w* ^*1118*^ and *Ore-R* are controls, *L70* has an insertion located at the 5’ of the *miR-310* ^*c*^, *F1*.*1* is a revertant line, where the P-element insertion of *L70* line has been precisely excised. *L4* line has an insertion in *esg*. *L70* and *L4* showed nicotine hypersensitivity. (C) Schematic representation of the *L4* P{GawB} insertion. The triangle represents the insertion site according to fly-base standard genome coordinates (release version 6). Zinc fingers are denoted by orange bands, the site of the *esg* ^*35ce-3*^ mutation is shown by an arrow. (D) Schematic representation of the *L70* P{GawB} insertion. The inverted triangle represents the insertion site according to fly-base standard genome coordinates (release version 6). The arrow shows the *miR-310* ^*c*^ cistron transcription initiation site. (E) *L70* and *L4* insertion lines are sensitive to chronic nicotine exposure. Restricted mean lifespan of mutant genotypes are significantly reduced compared to *w* ^*1118*^ when maintained in standard cornmeal food, supplemented with 0.5 mg/ml nicotine. All experiments were repeated at least 3 times with an n ≥ 100 flies. * = P≤ 0.01, ** = P≤ 0.001, **** = P<< 0.001, ns = not significant.

Based on McClung and Hirsh [5], we isolated mutants that have a differential response to nicotine compared to control lines, *white* ^*1118*^ *(w* ^*1118*^) and *Oregon-R* (*Ore-R*) flies. When flies are exposed to nicotine, they lose their negative geotaxis reflex for a period of time that is proportional to the amount of vaporized nicotine. In order to determine the standard amount of nicotine to be used, we treated *w* ^*1118*^ flies with different quantities of nicotine diluted in water to identify the sufficient amount of volatilized nicotine that allows half of the exposed flies to recover in 30 min. This parameter is defined as the Half Recovery Time (HRT). In our conditions *w* ^*1118*^ has a HRT of 30 min when exposed to 32 ng of nicotine. All further experiments were performed using this amount of nicotine. Line *w* ^*1118*^ was used as control and reference line, because it is the genetic background of the *P{GawB}* insertion collection and of all the other lines used in this work, *Oregon-R* was also used as an independent wt line and behaved identically to *w* ^*1118*^ thus strongly suggesting that this is the wt sensitivity to nicotine (Fig 1A). A collection of 200 lines with random *P{GawB}* insertions in their genome was screened, and from this process we isolated two lines, *L4* and *L70*, which are hypersensitive to nicotine exposure. None of the other insertion lines tested showed any statistically significant differential sensitivity to nicotine when compared to the control lines. Heterozygous individuals of both lines, *L4*/+ and *L70*/+, have an augmented HRT of 71 and 52 min, respectively, compared to *w* ^*1118*^ (30 min). Homozygous *L70* is even more sensitive having a HRT of 145 min, suggesting a robust dosage effect (Fig 1B), while homozygous *L4* is lethal. The mock exposure of *L4* and *L70* to volatilized water (the nicotine vehicle) had no effect on their climbing capacity when compared with controls, demonstrating that the longer HRT is due to higher nicotine sensitivity and not to motility impairment (S1 Fig).

The insertion sites of *P{GawB}* in these mutants were identified using inverse PCR (*L70*) and plasmid rescue (*L4*) and confirmed by PCR amplification and sequencing of the insertion site. *L4* insertion site is 39 nucleotides downstream of the transcription initiation site of the *escargot* gene (*esg*) (Fig 1C). *L70* is homozygous viable and fertile and its insertion site is 10 nucleotides upstream of the transcription initiation site of the *miR-310* ^*c*^ (Fig 1D). To demonstrate that the *P{GawB}* insertion causes hypersensitivity in *L70*, five independent precise excision lines were isolated by mobilizing the *P{GawB}* element. All these reverted lines showed normal sensitivity to nicotine (Fig 1B and S2 Fig).

In order to measure if the exposure route would have an effect on the sensitivity, and if the phenotype is different depending on an acute or chronic exposure, we exposed the flies to food containing 0.5 mg/ml of nicotine and measured their mean restricted lifespan. As shown in Fig 1E, *L70/+* and *L4/+* are significantly more sensitive to nicotine-containing food than control flies (*w* ^*1118*^ 16.98 ± 0.41 days, *Ore-R* 16.87 ± 0.45 days, *L70/+* 9.07 ± 0.61 days, *L4/+* 9.68 ± 0.86 days, *L70/L70* 1.77 ± 0.09 days). Therefore, these lines are hypersensitive to nicotine regardless of the way they are exposed to it (volatilized or ingested).

### *L4* is an *escargot* loss-of-function mutation and causes nicotine hypersensitivity

**Fig 2:**
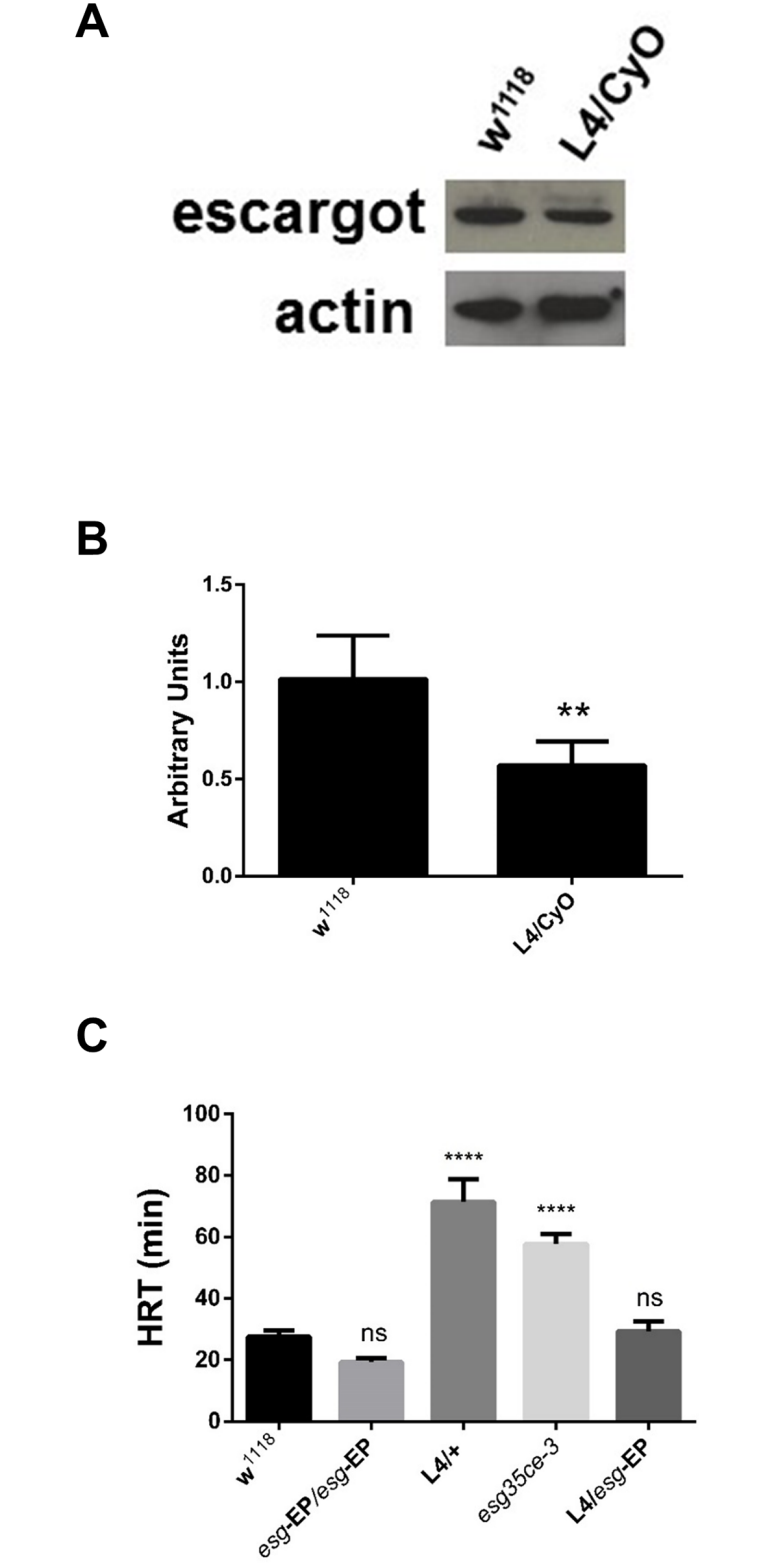
*L4* P-element insertion causes Esg loss of function. (A) Western blot of *L4* embryos showing a reduced expression of the Esg protein compared to *w* ^*1118*^. (B) Quantitative densitometry of western blots. n = 3 (C) Half recuperation time (HRT) of the different *esg* alleles used. EP = *P{EP}esg* ^*EU143*^, *esg* ^*35ce-3*^ (loss of function allele) ** = P≤ 0.001, **** = P<< 0.001.

To evaluate how the P-element insertion was affecting Esg levels in the *L4/+* line, we performed western blot analysis using embryos because *esg* expression levels are reported to be highest at this developmental stage [6]. We found that in embryos, *L4* has approximately half of Esg protein compared to the control line *w* ^*1118*^ (Fig 2A and 2B). We attempted to perform western blots of adult extracts but Esg could not be detected in these conditions. This result is consistent with reports showing a very low *esg* expression at this stage [7,8]. Additionally, we found that in approximately 8% of the population, *L4* flies have the characteristic cuticle defects reported in other *esg* loss of function allelles, such as loss of bristles in the tergites and sternites, and loss of pigmentation in the dorsal abdomen [7]. These data strongly suggest that *L4* leads to a loss of function of *esg*.

In order to verify if the loss of function of *esg* is causing hypersensitivity to nicotine, we tested another *esg* mutant allele (*esg* ^*35Ce-3*^), that produces an inactive transcription factor due to a point mutation in the third Esg zinc-finger domain [8]. This allele also has a homozygous lethal phenotype and the sensitivity of heterozygous individuals to nicotine is almost identical to *L4* heterozygous individuals (Fig 2C). In addition, the transheterozygous genotype *L4/esg* ^*35Ce-3*^ is lethal, therefore confirming that *L4* is an *esg* loss of function allele. *L4* is an enhancer trap insertion as it carries GA*L4* in the *P{GawB}* element, driving the expression of any gene under the control of UAS sequences in the endogenous *esg* expression pattern. The EP insertion *P{EP}esg* ^*EU143*^ can be used to express *esg* under the control of any GAL4 driver. Recovering the *esg* expression levels in the *L4/ P{EP}esg* ^*EU143*^ (*L4> P{EP}esg* ^*EU143*^) genotype led to normal sensitivity to nicotine (Fig 2C).

These results clearly indicate that a reduction of one half of the *esg* gene dosage is sufficient to reproduce the nicotine hyper-sensitive phenotype.

### The *L70* insertion causes the *mirR-310-311-312-313 cluster* (*miR-310* ^*c*^) to be miss-expressed

**Fig 3:**
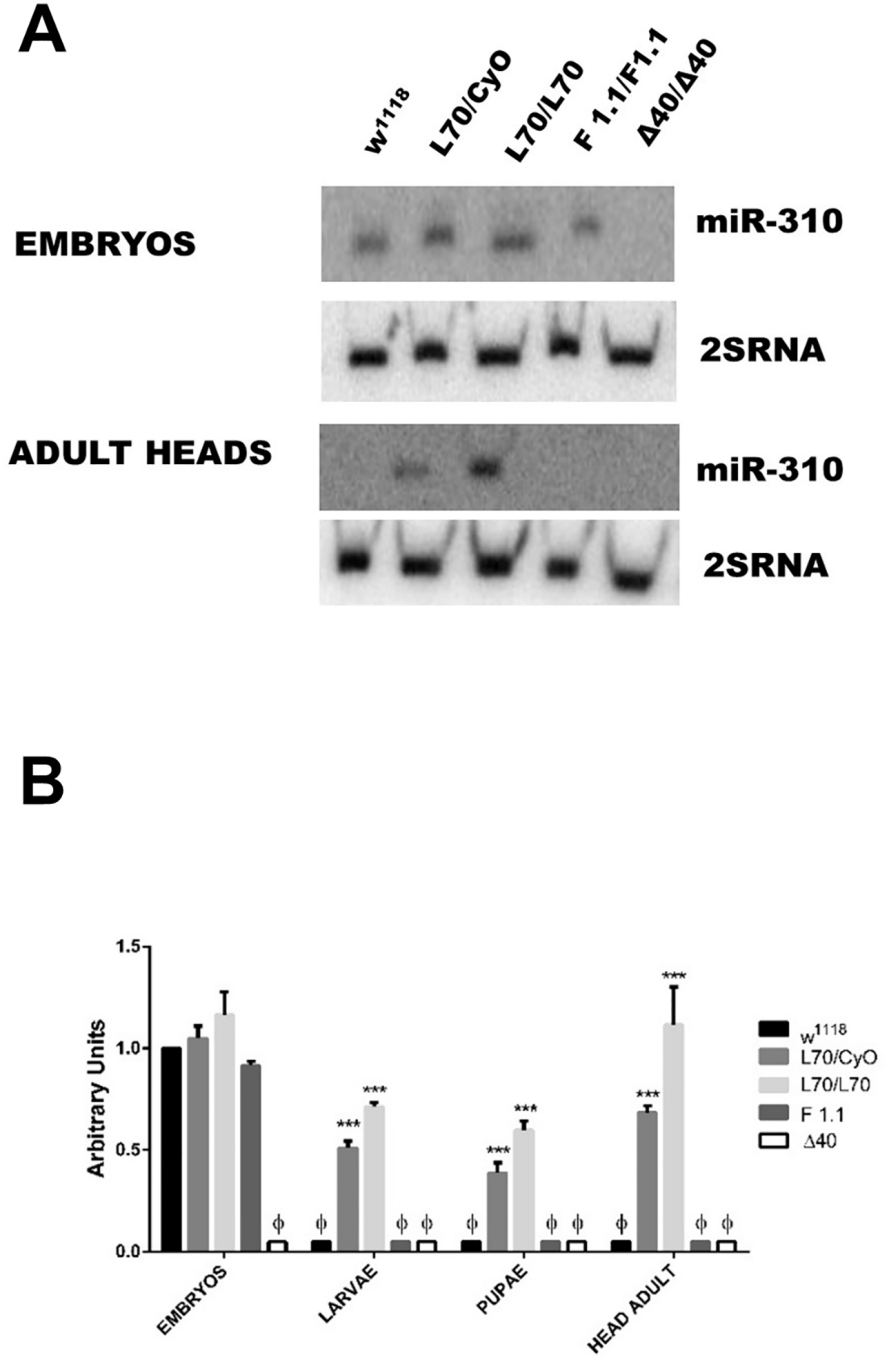
*L70* P-element insertion causes ectopic expression of the *miR-310* ^*c*^ in adult flies. (A) Northern blots of fly embryos and adult heads show that there is no adult expression of the miR-310 transcript in adult tissues in *w* ^*1118*^ and revertant line *F1*.*1* while there is expression of the miR-310 transcript in *L70*. Δ40 strain has a deletion of the complete *miR-310* ^*c*^ and is used as a negative control. Similar results were found with the other miRs transcripts (S2 Fig). (B) Densitometric analysis of (A). Ф = not detectable, *** = P≤ 0.0001.

To evaluate how the P-element insertion was affecting the expression levels of the *miR-310* ^*c*^ in *L70* line, we performed northern blot analyses using embryos, larvae, pupas and adult flies. We found no difference between *L70* and *w* ^*1118*^ embryos. However, the *miR-310* ^*c*^ was expressed both in *L70* adults and pupae whereas no expression, or extremely low expression, was detected in in the control line, *w* ^*1118*^, at these stages (Fig 3A and 3B and S3 Fig). It has been reported that the *miR-310* ^*c*^ is strongly expressed during early embryo development (0–12 hrs), with its expression decreasing in 12 to 24 hrs embryos and being extremely low in larvae, pupae and adults [9]. These data indicate that the *L70* insertion caused somehow a deregulation of the *miR-310* ^*c*^ expression, provoking its ectopic expression at late developmental stages. As the insertion site of *P{GawB}* element was close to the transcription initiation site (10 bp), we presumed that the transcription initiation of this gene could be affected; however, by 5'RACE, we corroborated that the *miR-310* ^*c*^ transcription initiation site in *L70* adults is identical to the transcription initiation site of wt embryos (S4 Fig).

### Nicotine sensitivity of *L4* and *L70* is not due to a general lack of fitness

**Fig 4:**
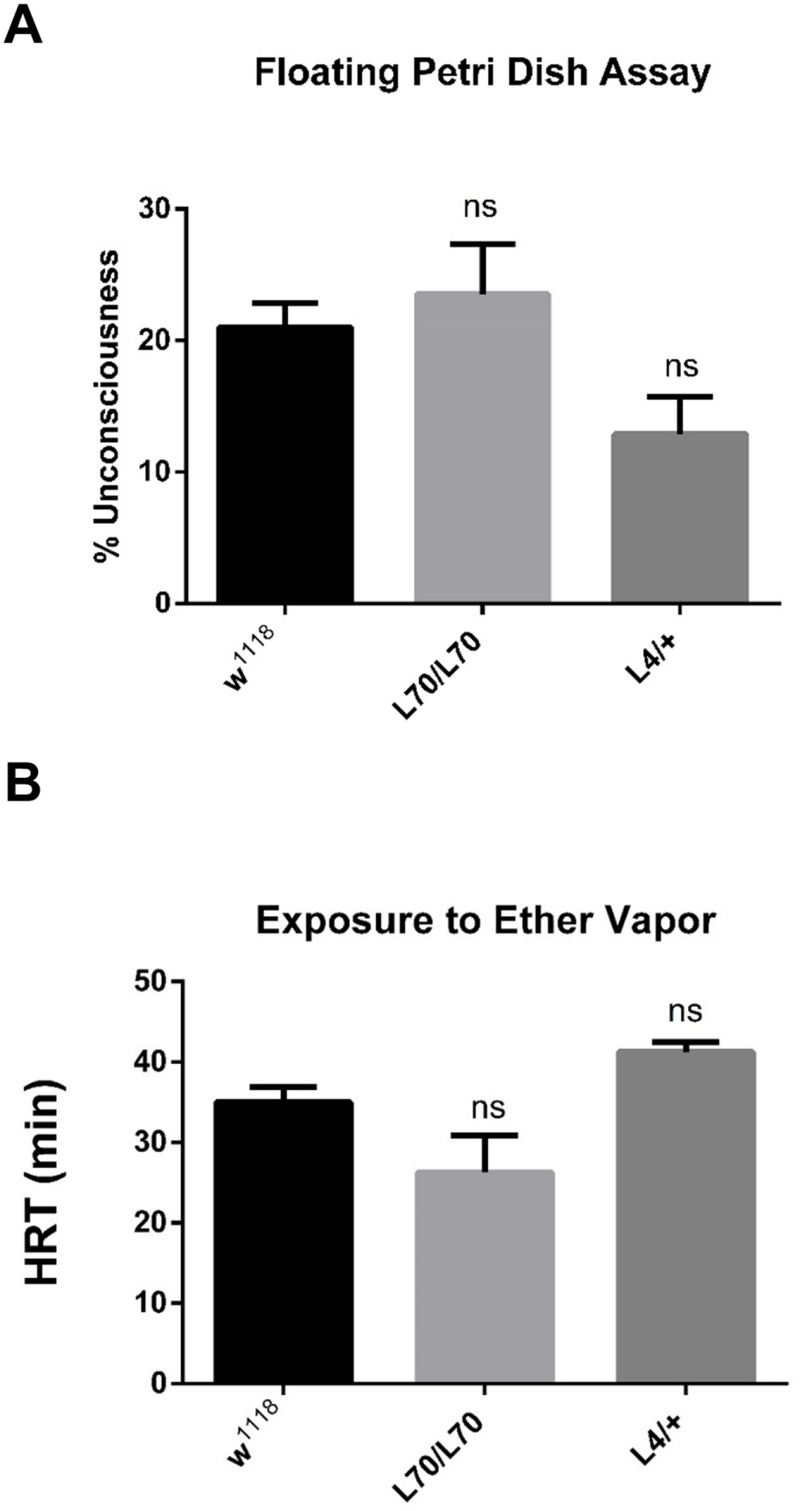
*L70* and *L4* mutant lines are not hypersensitive to heat or ether. (A) Percentage of unconscious flies exposed to 45°C in the floating plate assay: *L70* and *L4* are as resistant to heat as *w* ^*1118*^. (B) HRT of L4 and L70 flies exposed to ether. No significant (ns) difference was detected in either condition.

To evaluate the possibility that *L4* and *L70* nicotine hypersensitivity is due a general lack of fitness, we performed tests with different environmental factors that may uncover some other type of fitness defects. Heat resistance and ether sensibility can be used as proxy of general fitness. Heat resistance can be evaluated using the floating Petri dish assay (see Materials and methods). Our data showed that *L4* and *L70* contend normally with heat (Fig 4A). The ether sensibility of *L4* and *L70* was also normal (Fig 4B). These data together with the fact that *L4* and *L70* flies have the same climbing abilities than the controls (S1 Fig) suggest that these mutant lines do not have a general lack of fitness.

### Expressing small interference RNAs against *esg* or expressing the *miR-310* ^*c*^ under the control of *L4* enhances the mutant *esg* cuticle phenotype and nicotine sensitivity

**Fig 5:**
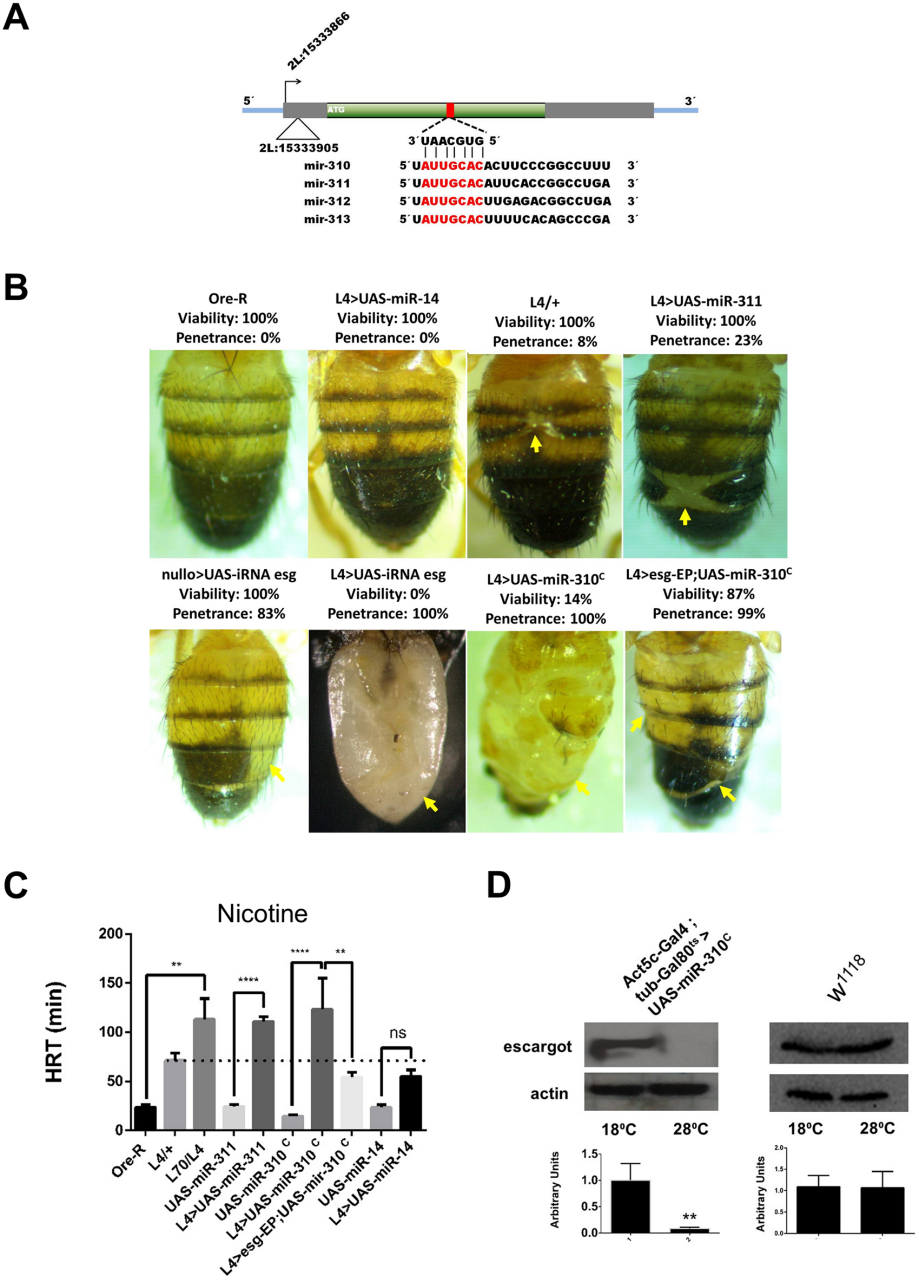
Genetic interaction of the *miR-310* ^*c*^ with *esg*. (A) Schematic representation of *esg* gene. The red rectangle represents the target sequences in *esg’s* ORF of miR-310, miR-311, miR-312 and miR-313. An alignment of the four mRNAs encoded in the *miR-310* ^*c*^ is also shown, red characters show the seed sequence. (B) Phenotype penetrance of the *L4* flies expressing small RNAs: *Ore-R* = wt abdomen, *L4*> UAS-miR-14 = miR-14 driven by *L4* (note that there is no apparent phenotype) *L4/+* = basal *L4*, L4>UAS-miR-311 = miR311 driven by *L4*, nullo>UAS-iRNA esg = short interference RNA against *esg* driven by nullo, *L4*>UAS-iRNA esg = short interference RNA against *esg* driven by *L4*, *L4*>UAS- *miR-310* ^*c*^ = *miR-310* ^*c*^ driven by *L4*,*L4*>esg-EP;UAS-*miR-310* ^*c*^ = *miR-310* ^*c*^ and *esg* from the EP driven by *L4*. Phenotype of the small interference RNA against *esg* is similar to the miR-311 and *miR-310* ^*c*^ phenotype (cuticle phenotype is indicated with yellow arrows). Note that the presence of the esg-EP suppresses the effect of the *miR-310* ^*c*^ expression (L4>esg-EP;UAS-*miR-310* ^*c*^). (C) HRTs become significantly longer when *L70* and *L4*0 genetically interact (*L70*/*L4*). This interaction can be phenocopied by the *L4* induced expression of the *miR-310* ^*c*^ (*L4*> UAS- *miR-310* ^*c*^) or miR-311 alone, suggesting that the *miR-310* ^*c*^ down-regulates *esg* expression. Dashed line indicates the basal sensitivity of *L4/+*. n≥100, * = P ≤ 0.01, ** = P ≤ 0.001, **** = P<< 0.001, ns = not significant. (D) Western blots show that Esg protein levels can be conditionally ablated when the *miR-310* ^*c*^ is expressed. The *miR-310* ^*c*^ can only be expressed at 28°C in the Act5c-GAL4;Tub-Gal80^ts^>UAS-*miR-310* ^*c*^ genotype, in these conditions *esg* becomes undetectable, while in the control (*w* ^*1118*^) remains unaffected, lower panels correspond to the densitometric analysis of the western blots, ** = P≤ 0.001.

Analysis of the *esg* transcript using TargetScanFly 6.2 [10] showed that it contains one complementary target site for the seven nucleotide “seed” sequence shared by the four microRNAs in the *miR-310* ^*c*^. The target sequence is located in the 3’ end of *esg’s* open reading frame, from nucleotides 1113 to 1119 (Fig 5A). Interestingly, in the *Drosophila esg* homolog family members: *snail*, *worniu* and *scratch* this seed sequence is absent. We tested *snail* ^*18*^, a loss of function mutant, and it did not present any abnormal nicotine sensitivity thus suggesting that *esg* has a direct involvement in nicotine sensitivity and that the expression of the *miR-310* ^*c*^ down-regulates *esg*. Although animal canonical regulation by microRNAs involves targeting of the transcript 3´UTR, there are well-documented cases showing that microRNA regulation is also achieved by targeting the coding region, especially in *Drosophila*[11,12]. Noteworthy, it has been shown that the *miR-310* ^*c*^ regulates Khc-73 via a target sequence located in its ORF [13].

MicroRNA target regulation is usually demonstrated using artificial constructs that have the microRNA target sequence fused to luciferase and measuring *in vitro* the reduction in luciferase activity. We are fortunate to have an *in vivo* and biologically relevant phenotypic reporter that is the classic loss of function *esg* phenotype. As mentioned above, loss of function of *esg* in the ectoderm during development causes characteristic cuticle defects whose extent is proportional to the amount of *esg* loss of function [7]. In order to demonstrate that cuticle defects can be used as a reporter of *esg* loss of function, we used RNA interference to down-regulate *esg* using the *nullo* promoter that is a low level constitutive driver which is expressed in all somatic tissues [14]. In this genotype (*nullo>UAS-iRNA-esg*) the cuticle phenotype is induced in 89% of the flies with 100% viability. Furthermore, when *L4* is used to drive the *UAS-iRNA-esg* construct (*L4>UAS-iRNA-esg*), the cuticle phenotype penetrance is enhanced from 8%, in *L4* by itself, to 100%. In this genotype, the *esg* phenotype is so severe that flies are unable to eclose and have to be dissected from the pupal case to evaluate cuticle damage (Fig 5B).

When the *miR-310* ^*c*^ is expressed from the UAS-DsRed53-*mir-310-313* line [13] using *L4 as a* driver we found that the cuticle phenotype penetrance increased to 100%, although it was less severe than the one induced by the *UAS-iRNA-esg*, as they have 14% of viability. The eclosed flies behaved normally but were as sensitive to nicotine as the *L4/L70* flies (Fig 5C). These data suggest that *miR-310* ^*c*^ is lowering Esg levels in a similar manner as the *esg* iRNA. This is also supported by the fact that individually expressed miR-311 has an enhanced cuticle phenotype with a penetrance of 23% with 100% viability (Fig 5B). The expression of the non-related microRNA *miR-14* driven by *L4* had no effect on nicotine HRT or cuticle phenotype (Fig 5B).

To confirm that the cuticle phenotype induced by the expression of the *miR-310* ^*c*^ is specifically caused by a reduction of *esg* function, this phenotype was reverted by augmenting the *esg* gene dosage using the following genotype: *L4*>esg-EP;UAS-*miR-310* ^*c*^. In these flies, cuticle defects were partially rescued and viability increased from 14% to 87% (Fig 5B). Nicotine sensitivity was rescued as the HRT of this line decreased from 123 minutes, shown by the L4>UAS-*miR-310* ^*c*^, to 54 minutes in *L4*>esg-EP;UAS-*miR-310* ^*c*^ (Fig 5C).

To further demonstrate that the *miR-310* ^*c*^ overexpression reduces the amount of Esg, we performed a western blot analysis of embryos having the following genotype: Act5c-GAL*4*;tub-GAL80^ts^>*miR-310* ^*c*^. Embryos were used because in adult flies Esg levels are extremely low. The expression of this cluster during embryo development reduces *esg* protein to undetectable levels thus explaining the lethality observed when the *miR-310* ^*c*^ is expressed early in development with a strong promoter (Fig 5D). Overall, these experiments clearly demonstrate that the *miR-310* ^*c*^ is downregulating *esg*.

### The phenotype of the ectopic expression of the *miR-310* ^*c*^ can be phenocopied by a constitutive GAL4 driver but only when it is expressed late in development

**Fig 6:**
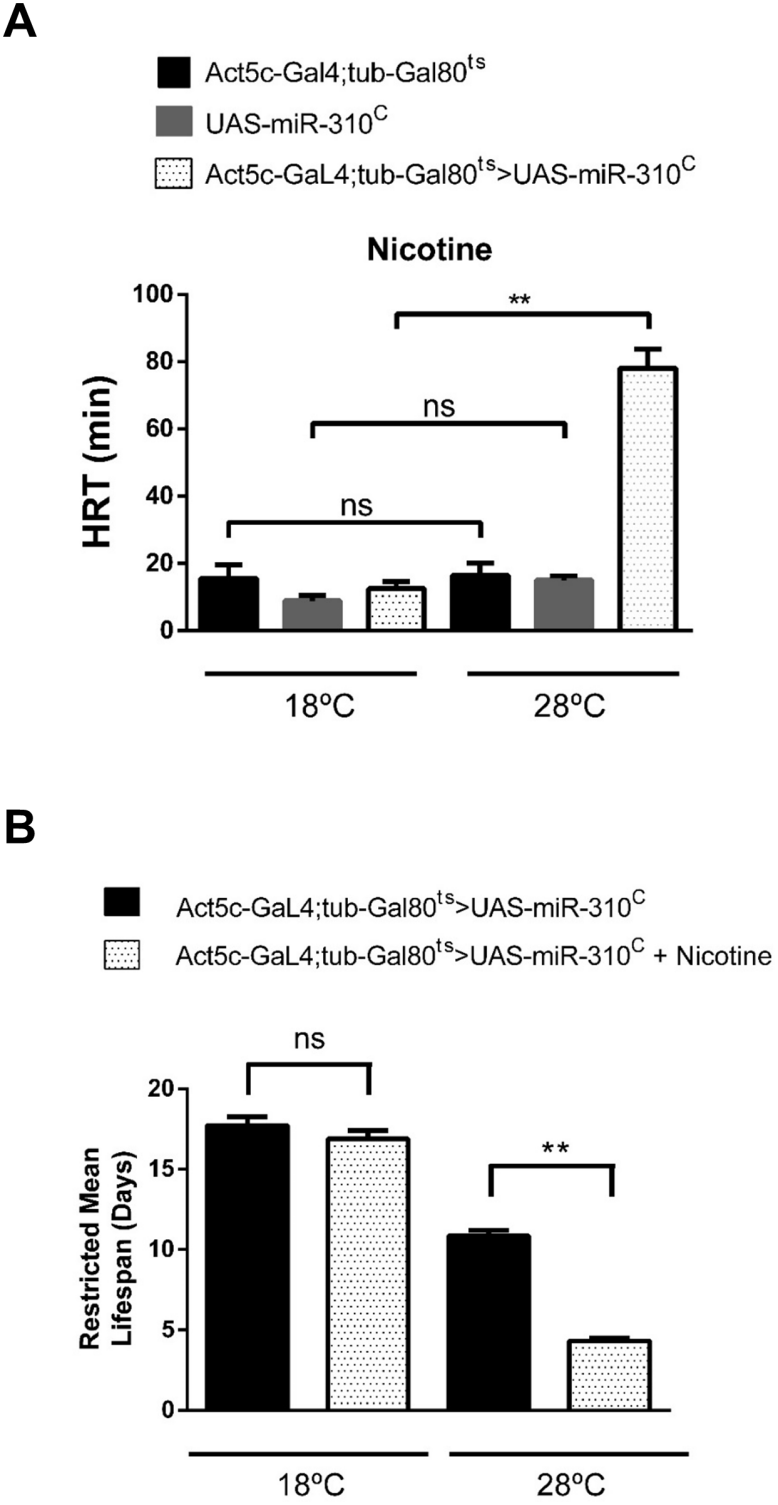
Temporally controlled expression of the *miR-310* ^*c*^ in adult flies phenocopies *L70* mutant line. (A) The HRT of the line Act5c-GAL4;Tub-Gal80^ts^>UAS-*miR-310* ^*c*^ is significantly longer only when it is at the GAL80 permissive temperature (28°C). Embryos were reared at 18°C for sixteen days, just before eclosion pupae were transferred to 28°C and maintained at this temperature for 3 days before assaying, controls were kept at 18°C, n ≥ 100. (B) Survival of the line Act5c-GAL4;Tub-Gal80^ts^>UAS-*miR-310* ^*c*^ is significantly reduced. Restricted mean life of Act5c-GAL4;Tub-Gal80^ts^>UAS-*miR-310* ^*c*^ genotype is significantly reduced when maintained in standard cornmeal food supplemented with 0.5 mg/ml nicotine at the GAL80 permissive temperature n ≥ 100 flies, n = 3 in all experiments, ** = P ≤ 0.001, ns = not significant.

To demonstrate that the ectopic expression of the *miR-310* ^*c*^ is enough to enhance the nicotine-sensitivity, we used the transgenic UAS-DsRed53-*mir-310-313* line driven by a battery of GA*L4* drivers of different strengths and directed to different target organs; however, no driver was able to direct the expression of the *miR-310* ^*c*^ in such a way that it phenocopies *L70* (S1 Table). A possible explanation for this result could be that the *miR-310* ^*c*^ expression was not directed to the correct tissues, time and/or levels of expression. We attempted to drive the expression of the *miR-310* ^*c*^ by strong constitutive GA*L4* drivers such as the actin promoter but we only confirmed that constitutive strong expression of the microRNAs is lethal as previously reported (S1 Table) [15]. To overcome this issue, we decided to use the GA*L4*/GAL80^ts^ system in order to control the time at which the *miR-310* ^*c*^ was expressed. For this, we induced the *miR-310* ^*c*^ expression by incubating flies at 28°C, the permissive temperature, at different times during development using the Act5c-GA*L4*;tub-Gal80^ts^ driver. Under these conditions, flies were viable (60%) only when the expression of the *miR-310* ^*c*^ was induced after sixteen days of development at 18°C when they are just at the end of pupation or after eclosion. Flies expressing *miR-310* ^*c*^ became as sensitive to nicotine as the *L70* line after 72 hrs of induction (Fig 6A). Also, these flies are more sensitive to nicotine-containing food thus fully phenocoping *L70* (Fig 6B).

### The gene *esg* is expressed in labial imaginal discs

**Fig 7:**
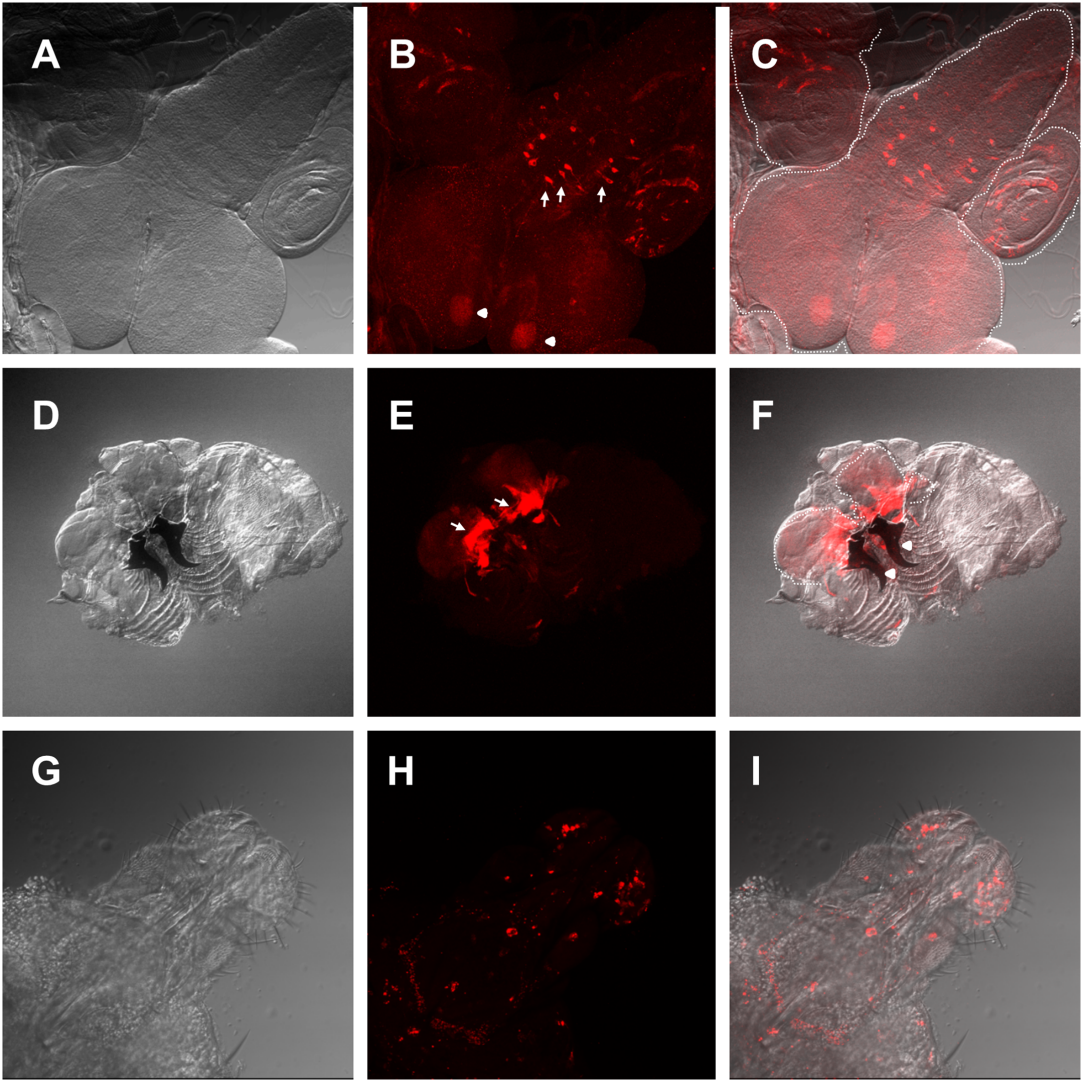
Expression pattern of the *esg L4* enhancer trap. (A-C) L4>UAS-GFP third instar larval central nervous system (CNS) and adjacent imaginal discs A) Differential interference contrast (DIC) microscopy image. (B) Confocal maximal projection of an immunohistochemistry against GFP. Arrows show the medial section of the thoracoabdominal ganglion *esg* expressing neurons and arrowheads show mushroom bodies. (C) Overlay of DIC and confocal images. (D-F) L4>UAS-GFP third instar larval inverted maxillary region that includes labial imaginal discs. (D) DIC microscopy image. (E) Confocal maximal projection of GFP fluorescence. Arrows show the expression domain in the proximal labial discs. Arrowheads show mandibles. (F) Overlay of (D) and (E). (G-I) L4>UAS-Stinger late pupal proboscis. (G) DIC microscopy image. (H) Confocal maximal projection of stringer fluorescence. (I) Overlay of (H) and (I). White dash-lines show the border of the CNS and labial imaginal discs, respectively.

Trying to understand the cellular basis of these phenotypes, we characterized the *L4* expression pattern by crossing it with UAS-GFP and found that it has the same expression pattern as the one reported for *esg*: embryonic ectoderm and histoblasts; larval leg and wing imaginal discs, brain, spiracles and tracheae; adult intestinal stem cells, malpighian tubules, testis and salivary glands [7,16–19]. Our data shows that *esg* is also expressed in the third instar larval mushroom bodies and the medial section of the thoracoabdominal ganglion, where motor neurons that innervate wings and legs are located. (Fig 7A–7C). Importantly, we found that *esg* is expressed in the proximal region of the third instar larvae labial imaginal discs (Fig 7D–7F) and in the labial palp of the late pupae (Fig 7G–7I) where the taste organs are developing. The lack of *esg* may be altering the normal differentiation of these organs causing hypersensitivity to nicotine.

### Transcriptome analysis shows that *esg* loss of function affects genes involved in chemical perception and cuticle development

We sequenced the transcriptome of *L4* and *L70* heads and compared each of the mutant lines with both control lines (*w* ^*1118*^ and *Ore-R*). To identify the biological processes altered in our mutants, we used the resources of the Gene Ontology Consortium [20,21] to do an enrichment of biological process analysis, using the database of genes whose ontology is supported by experimental data. For this analysis, we only considered genes that were consistently up or downregulated in the mutant lines when compared with both control lines. We used as cutoff a “double control log2 ratio” (see Materials and methods) of 1 or greater, that is equivalent to a minimum of a 2 fold increase when compared to the controls’ levels of expression; or -1 or lower, that is equivalent to a minimum reduction of half of the controls’ levels of expression. Computed genes with no mutant phenotype, molecular feature or determined function (CGs followed by an integer) were not included. Using these criteria, we found that in *L4*, 507 genes were upregulated and 233 were downregulated. The biological processes in which the affected genes in *L4* participate are related to male gamete generation, cuticle development and detection of chemical stimulus: odorant, pheromones, taste and chemical receptors (S2 Table). For *L70*, 164 genes were upregulated while 233 were downregulated. The affected genes in *L70* are involved in the following biological processes: detection of chemical stimulus involved in sensory perception (specifically smell), neurological system process, detection of pheromones, sexual reproduction and nervous system development (S3 Table). These data are in agreement with our observed phenotypes and also with previous literature as both *esg* and the *miR-310* ^*c*^ had been reported to be required for male gametogenesis and neural development [22,23].

## Discussion

In this work, we identified two mutant lines that are statistically significant more sensitive to nicotine when compared to control flies. One of them is *L4*, a loss of function allele that reduces the levels of the transcription factor Esg by half, as demonstrated by western blot analysis. The hypersensitivity of this P-element insertion mutant is clearly reproduced by *esg* ^*35Ce-3*^, a loss-of-function mutant that expresses a function-less full length Esg protein with one of its zinc fingers inactivated by a missense point mutation. This loss of function is further confirmed by the fact that the *L4/esg* ^*35Ce-3*^ genotype is lethal, demonstrating that they belong to the same complementation group. In addition, the induced expression of *esg* by *L4* using an EP insertion in the *esg* gene completely rescues the normal nicotine phenotype; thus, confirming that the loss of half the dosage of *esg* is enough to confer hypersensitivity to nicotine.

*L4* shows the classical *esg* cuticle phenotype previously reported for other *esg* alleles. The *esg* knock-down obtained by RNAi severely enhances cuticle phenotype, in the same way as the miss-expression of the *miR-310* ^*c*^ does, either by its own promoter or by using the nullo driver, consequently demonstrating that this cluster is down-regulating the *esg* gene. The *esg* downregulation was also shown in embryos, where the *miR-310* ^*c*^ over expression completely abates Esg production.

We found *esg* expression in the proximal labial imaginal disc, the precursor of the proboscis, where the main gustatory organs reside. In order to explain the nicotine hypersensitivity we propose that the loss of *esg* function in the labial discs during development is promoting premature differentiation of these sensory organ precursors affecting their cellular fate and causing ectopic expression of chemical receptors. Esg, and the two other members of the family, Worniu and Snail, participate in neuroblast development and differentiation by mediating asymmetric cell divisions that has been shown to be involved in the correct development of sensory bristles and other taste specialized sensilla [24,25]. Esg affects aspects of the differentiation process in intestinal stem cells and male germ cells by maintaining stemness and repressing differentiation [26]. This Esg function might be conserved during gustatory organ development and differentiation and the development of neurons of the medial section of the thoracoabdominal ganglion. This hypothesis is supported by the fact that other Snail family members have a similar role in the nervous system [27,28], and by our sequencing data that showed that many genes involved in sensory and chemical perception are affected when there is *esg* loss of function. The change in cell fate hypothesis that we propose is further supported by the fact that *esg* is involved in olfactory neuron fate determination as the over-expression of Esg together with Nerfin induces the appearance of CO_2_ sensitive neurons that do not exist in the wild type [29]. The fact that that *esg* is expressed in the labial discs, proboscis and labella and that, in our transcriptome analysis, the expression of several bitter taste and odorant receptors were affected is consistent with an aberrant chemical sensilla differentiation caused by the lowered expression of *esg*, either through its downregulation via miRs or by mutation. The lack of *esg* could also be affecting other sections of the nervous system such as the medial section of the thoracoabdominal ganglion, where many of the motor neurons that control limb movement are located, thus contributing to the loss of negative geotaxis reflex of *esg* mutants when exposed to nicotine.

The other nicotine hypersensitive line, *L70*, showed ectopic expression of the *miR-310* ^*c*^ during pupation and in adult flies when it is not normally expressed. This altered *miR-310* ^*c*^ expression is sufficient to induce nicotine hypersensitivity, as demonstrated by ectopically expressing the *miR-310* ^c^ late in pupal development under the control of a strong constitutive promoter. The hypersensitivity shown by *L70* can be explained if the ectopic expression of these micro-RNAs downregulates *esg*, thus phenocopying *esg* loss of function. The stronger phenotype observed in homozygous *L70* compared to *L4* can be explained by the fact that the miRs in this cluster have additional target genes that affect neural physiology. The known targets of the *miR-310* ^*c*^ are β-catenin, dTCF [22] and Khc-73 [13]. Khc-73 is important for the modulation of synaptic strength, so its downregulation could be altering the intensity of the response to nicotine; βcatenin and TCF could be involved in neuron determination and synapse physiology via the Wg/Wnt pathway.

It is worth to mention that our work does not exclude metabolic pathways involved in detoxification, a line of inquiry that must be pursued in the future. We need to further investigate the precise levels of Esg during metamorphosis in these lines and to analyze in greater detail both the architecture and physiology of the adult nervous system in these lines.

In conclusion, we show that *esg* is important for the development of organs involved in chemical perception and that the ectopic expression of the *miR-310* ^*c*^ modulates *esg* expression.

## Materials and Methods

### Volatilized nicotine sensitivity test

Groups of 10 male flies 0–3 days post-eclosion were put into an empty vial (VWR vial O.D. x H = 25 x 95 mm), where they were exposed for 15 seconds to volatilized nicotine (N3976 SIGMA-ALDRICH). Nicotine solution was volatilized by placing 2 μl on a 3.0 mm Nichrome filament (NI30PF World Precision Instruments, Inc.), which was introduced into the vial and heated to 250°C to achieve instantaneous volatilization. To titrate the amount of nicotine to be used 0 to 1000 ng of nicotine were tested. The flies were then transferred into a clean vial labeled with a perimetral line 2.5 cm from the bottom. The negative geotaxis reflex was measured every 5 minutes. Flies that crossed the line in a time no longer than 10 seconds after being tapped to the bottom were considered recovered and removed from the vial. The observation time was performed until 100% of the flies recovered. Half Recovery Time (HRT) was defined as the time at which 50% of the flies recovered their geotaxis reflex. The amount of 32 ng of nicotine in 2 μl caused an HRT of approximately 30 minutes for the line *w* ^*1118*^; therefore, we used this amount in the rest of the tests. For statistical analysis of the data one-way ANOVA was used and a P ≤ 0.01 was considered significant. All graphs and statistical analyses were done in GraphPad Prism 6.01.

### Chronic exposure to nicotine

Newly eclosed male flies were placed in standard corn meal agar food (control) or with nicotine (experimental) at a concentration of 0.5 mg/ml in groups of 20–30 flies. Flies were changed every 24 hours to a new vial and the number of dead flies was registered until there were no more flies alive. Assays were performed at 18°C, 25°C and 28°C. For survival analysis, we used the Kaplan-Meier estimator using the OASIS: online application for the survival analysis of lifespan assays performed in aging research (http://sbi.postech.ac.kr/oasis/) [30].

### Floating Petri dish assay

The avoidance of noxious heat assay or hot water floating Petri dish assay was performed by introducing 30 adult male flies in a 14 cm plastic Petri dish sealed with vinyl electric tape. Petri dishes were floated in a 45°C water bath for 30 min. in darkness. Fit flies migrate to the roof of the petri dish and avoid becoming unconscious because of the heat shock, flies found unconscious were considered unfit [31]. For statistical analysis of the data one-way ANOVA was used and P ≤ 0.01 was considered significant. Graphs and statistical analyses were done in GraphPad Prism 6.01.

### Exposure to ether vapors

Groups of ten flies were placed for one minute in an empty standard *Drosophila* vial covered with a cotton plug that had 1 ml of ether. Flies were transferred to a fresh vial and the HRT was determined. The experiment was repeated at least ten times. For statistical analysis of the data one-way ANOVA was used and P ≤ 0.01 was considered significant. Graphs and statistical analyses were done in GraphPad Prism 6.01.

### Mapping of L70 and L4 mutations

*L70* mutant line was mapped using inverse PCR [32]. PstI and XbaI enzymes were used (NEB, Inc.) to digest genomic DNA. DNA was ligated with T4 ligase and fragments were amplified with the following oligonucleotides: F5'GCACGTTTGCTTGTTGAGAGGAAAGG3’ and R5'CGGTAAGCTAGCTTCGGCTATCG3'. The products obtained were sequenced and subsequently mapped to the *Drosophila* genome using BLAST analysis in http://flybase.org/blast/. The insertion site was mapped to 2R:20584338. *L4* mutant line was mapped by plasmid rescue technique [32,33]. PstI enzyme (NEB, Inc.) was used to digest genomic DNA and obtain pBluescript fused to adjacent genomic DNA. Three clones were obtained and sequenced using the T7 primer 5´TAATACGACTCACTATAGGG3’. Sequences obtained were identical and subsequently mapped to the *Drosophila* genome using BLAST analysis in http://flybase.org/blast/. The insertion mapped to site 2L: 15333905. Insertion sites were confirmed by PCR and the resulting products were sequenced. The oligonucleotides used were: 5'CACAACCTTTCCTCTCAACAA3' and 5'CTGACGTTGATCAGTTCCTCG3’ for the 5´ end of the *P{GawB}* insertion and oligos 5'TAGCCGAGTGCCGCGATTG3' and 5'CTGACGTTGATCAGTTCCTCG3’ for the 3' end.

### Northern-blot

Total RNA was prepared using Trizol reagent (Invitrogen, Carlsbad, CA), according to manufacturer’s directions. Total RNA (10–20 μg) was separated in a 15% poyacrylamide/7 M urea/0.5X TBE gel and transferred to Hybond-N^+^ membrane (Amersham, Piscataway, NJ). Hybridizations were carried out at 42°C with the Ultra-Hyb Oligo solution (Ambion, Austin, TX) using as probes DNA oligonucleotides labeled with P^32^-γATP (PerkinElmer) at their 5′ end using T4 polynucleotide kinase (Fermentas). Oligonucleotides used: miR-310-5’AAAGGCCGGGAAGTGTGCAATA3’, miR-311-5’tcaggccggtgaatgtgcaata3’, miR-312-5’tcaggccgtctcaagtgcaata3’, miR-313-5’tcgggctgtgaaaagtgcaata3’ and as a loading control 2SRNA-5´TACAACCCTCAACCATATGTAGTCCAAGCA. Two washes using 2x SSC/0.1% SDS were carried for 30 min at 42°C. Autoradiographs were analyzed using a Typhoon PhosphorImager (Amersham). All membranes were exposed for 24 hours and control hybridizations were exposed for 5 minutes.

## 5’ RACE

Total RNA was extracted from 3 day adult heads as above. 5´RACE was performed using the FirstChoice RLM-RACE Kit according to manufacturer´s instructions including a third round of nested PCR. Primers used:

First PCR: Del1Fwd 5´-GATTCGTTTTAAAGTAATCAGG-3´ and 5' RACE Outer Primer 5'-GCTGATGGCGATGAATGAACACTG-3'.

Second nested PCR: Del2Fwd 5´-GCTAATTGTTAGTATAAGGCA-3´ and 5' RACE Inner Primer 5'-CGCGGATCCGAACACTGCGTTTGCTGGCTTTGATG-3'.

Third nested PCR: miR-313 5´-TCGGGCTGTGAAAAGTGCAATA-3´ and 5' RACE Inner Primer 5'-CGCGGATCCGAACACTGCGTTTGCTGGCTTTGATG-3'.

PCR product was purified using Amicon Ultra-0.5 MILLIPORE columns and directly sequenced using primer miR-313.

### Western-blot

Total proteins from fly embryos aged 0–14 hrs were extracted in lysis buffer (250 mM sucrose, 50 mM Tris-HCl (pH 7.5), 5 mM EDTA, 25 mM KCl, 5 mM MgCl_2_ and 1% SDS) containing protease inhibitors. Protein extracts (50–100 μg) were subjected to 12% SDS-polyacrylamide gel electrophoresis, and transferred to Immobilon polyvinylidene difluoride membranes (MILLIPORE) for 2 hrs at 200 mA on a Mighty Small Transphor (Amersham Pharmacia Biotech). Blots were blocked overnight with 5% nonfat dry milk in PBS 1X containing 0.1% Tween-20. Custom made anti-Escargot antibodies (rabbit polyclonal, EZBiolab) were added at a dilution of 1:1000 for two hours and developed using the SuperSignal West Pico Chemiluminiscent Substrate (Thermo Scientific). After developing, blots were stripped by incubation at 55°C in a buffer containing: 62 mM TRIS-HCL pH = 6.8, 2% SDS and 0.1 M β-Mercaptoethanol. After stripping blots were assayed with anti-Actin antibody (Developmental Hybridoma Bank) using a dilution 1:3500 and developed as above. All blots were detected using Kodak radiographic film. Quantitation was performed using ImageJ [34] using the gel quantitation utility.

### Transcriptome analysis

Total RNA from fly male heads was extracted using Trizol reagent (Invitrogen, Carlsbad, CA), according to manufacturer’s directions. RNA was stabilized using RNA stable tubes from Biomatrica and processed according to manufacturer’s instructions. Fifty μg of total RNA per line were sent for deep sequencing to the Beijing Genome Institute (BGI Americas corporation). BGI provided the following services: 1. Data filtering that includes removing adaptors contamination and low-quality reads from raw reads. 2. Assessment of sequencing (Alignment Statistics, randomness assessment of sequencing, distribution of reads on the reference genome). 3. Gene expression and annotation (Gene coverage and coverage depth). 4. Gene expression difference analysis (this tables are available as provided by BGI in S4–S7 Tables) which were obtained with the NOIseq differential expression method [35]. Sample (L4 or L70) and control lines (*w* ^*1118*^ and *Ore-R*) were sequenced as independent duplicates. FPKMs for each duplicate were averaged and the mean log2 ratio was calculated using the following formula: (mean sample FPKM)/(mean control FPKM). Since we were using two different control strains, in order to avoid possible genetic background effects we specifically selected genes that were consistently up or down regulated in the sample when compared to both control lines. We obtained a “double control log2 ratio” by averaging the mean log2 ratios from both comparisons of each sample with both controls. Only genes that had a “double control log2ratio” bigger than 1 or smaller than -1 were selected for the gene ontology analysis. The genes that satisfied these requirements were analyzed with the tools from the Gene Ontology Consortium [20,21] to do an enrichment of biological process analysis, using the database of genes whose ontology is supported by experimental data. Raw data available at: http://www.ncbi.nlm.nih.gov/bioproject/288398. Accession numbers: SRR2080216, SRR2080214, SRR2080213, SRR2080215, SRR2080211, SRR2080210, SRR2080212.

### Immunohistochemistry

Tissue was dissected in PBS and fixed in PBS 4% formaldehyde. Tissue was then permeabilized with 0.2% TritonX-100 and blocked for 30 min with 5% goat preimmune serum at room temperature. The primary antibody (mouse anti-GFP, 1:10, developmental studies hybridoma bank) was added, incubated overnight at 4°C and washed 3 times in PBS 0.2% TritonX-100 at room temperature. The secondary antibody (Cy3conjugated, 1:300, goat anti-mouse, Rockland Immunochemicals, Inc., Gilbertsville,PA) was added and incubated for 90 min and washed 3 times in PBS 0.2% TritonX-100 at room temperature. Samples were mounted in Citifluor (Ted Pella Inc).

### Confocal Imaging

Images were collected at the “Laboratorio Nacional de Microscopía Avanzada (LNMA)” in an inverted Confocal multiphotonic Olympus FV1000, using optimal confocal pinhole. Images were analyzed and reconstructed using the open source ImageJ program [34].

### Stocks and crosses

Flies were raised on standard yeast medium at 18°C, 25°C and 28°C. *Oregon-R* and *w* ^*1118*^ strains were obtained from the Drosophila stock center in Bloomington, Indiana. *L4* (*w* ^*1118*^ *; P{GawB-GAL4*. *esg}* ^*L4*^) and L70 (*w* ^*1118*^ *; P{GawB-GAL4*. *miR310/313}* ^*L70*^), transgenic lines were obtained in our laboratory by mobilization of P-element P{GawB}. The P-element in *L70* was mobilized by crossing to the Δ2–3 transposase source. Revertant lines (F 1.1, F 1.2, F 2, F 5 and F 7.2) were recovered by screening for loss of the white marker, the insertion region was sequenced and revertant lines were tested for nicotine sensitivity. UAS-iRNA esg (Bloomington Stock # 34063), *snail* ^*18*^ (Bloomington Stock # 2311), Δ40 line (deletion of cluster *miR-310* ^*c*^), UAS-DsRed-53-miR-310-313 were kindly provided by Pejmun Haghighi Lab (McGill University), individual UAS-LUC-miR-311, UAS-LUC-miR-14 and all other lines used in this work are available at the Bloomington Drosophila Stock Center (NIH P40OD018537).

## Supporting Information

S1 Fig Response of flies to vaporized water. All mutant genotypes recovered immediately after exposure to vaporized water (nicotine 0 ng).
(TIF)

S2 Fig The precise excision of the P{GawB} revert *L70*’s hypersensitivity. P{GawB} was mobilized using the transposase source Δ2–3. Five independent lines: *F1*.*1*, *F1*.*2*, *F2*, *F5*, *F7*.*2* (data for *F1*.*1* is in Fig 1) were recovered, all of them having a precise excision that was confirmed by DNA sequence. HRT from all revertant lines did not show significant differences from the control lines. ns = not significant.
(TIF)

S3 Fig *L70* P-element insertion causes ectopic expression of the *miR-310* ^*c*^. (A) Northern blot and their quantitative analysis using RNA from embryos. (B) Northern blot and their quantitative analysis using RNA from adult male thorax. (C) Northern blot and their quantitative analysis using RNA from adult male abdomen. Probes used are indicated at the right of each hybridization result. There is practically no adult expression of the miR-310, miR-311 and miR-312 transcripts from wt, Δ40 and *F1*.*1* revertant line in adult tissues tested, while there is high expression of the transcripts in *L70* mutant line. miR-313 was not detectable. *** = P << 0.001, Ф = non detectable.
(TIF)

S4 Fig The transcription initiation sites of *miR-310* ^*c*^ in *w* ^*1118*^ and *L70* are identical. FirstChoice RLM-RACE Kit was used to determine the transcription initiation site of the miR-310c. A) Arrow shows third nested PCR product of *w* ^*1118*^ and sequence histogram. 1) MWM 2) Primers miR-313/Inner5´RACE, *w* ^*1118*^ cDNA. 3) Primers miR-313/Inner5´RACE, no cDNA B) Arrow shows third nested PCR product of *L70* and sequence histogram. 1) MWM 2) Primers miR-313/Inner5´RACE, *L70* cDNA. 3) Primers miR-313/Inner5´RACE, no cDNA C) Sequence alignment of w1118 and L70 PCR products.
(TIF)

S5 Fig Antibody Synthesis Report. (PDF)

S1 Table Drivers used for the expression of the *miR-310* ^*c*^. (DOCX)

S2 Table Gene onthology enrichment for *L4*. (DOCX)

S3 Table Gene onthology enrichment for *L70*. (DOCX)

S4 Table *w* ^*1118*^ vs *L4* gene differential expression. (XLS)

S5 Table *Ore-R* vs *L4* gene differential expression. (XLS)

S6 Table *w* ^*1118*^ vs *L70* gene differential expression. (XLS)

S7 Table *Ore-R* vs *L70* gene differential expression. (XLS)
