# Supplementary figures and images for: The Esg Gene Is Involved in Nicotine Sensitivity in Drosophila melanogaster
Source: PLoS One. 2015 Jul 29;10(7):e0133956. doi: 10.1371/journal.pone.0133956 (PMC4519288; doi:10.1371/journal.pone.0133956)

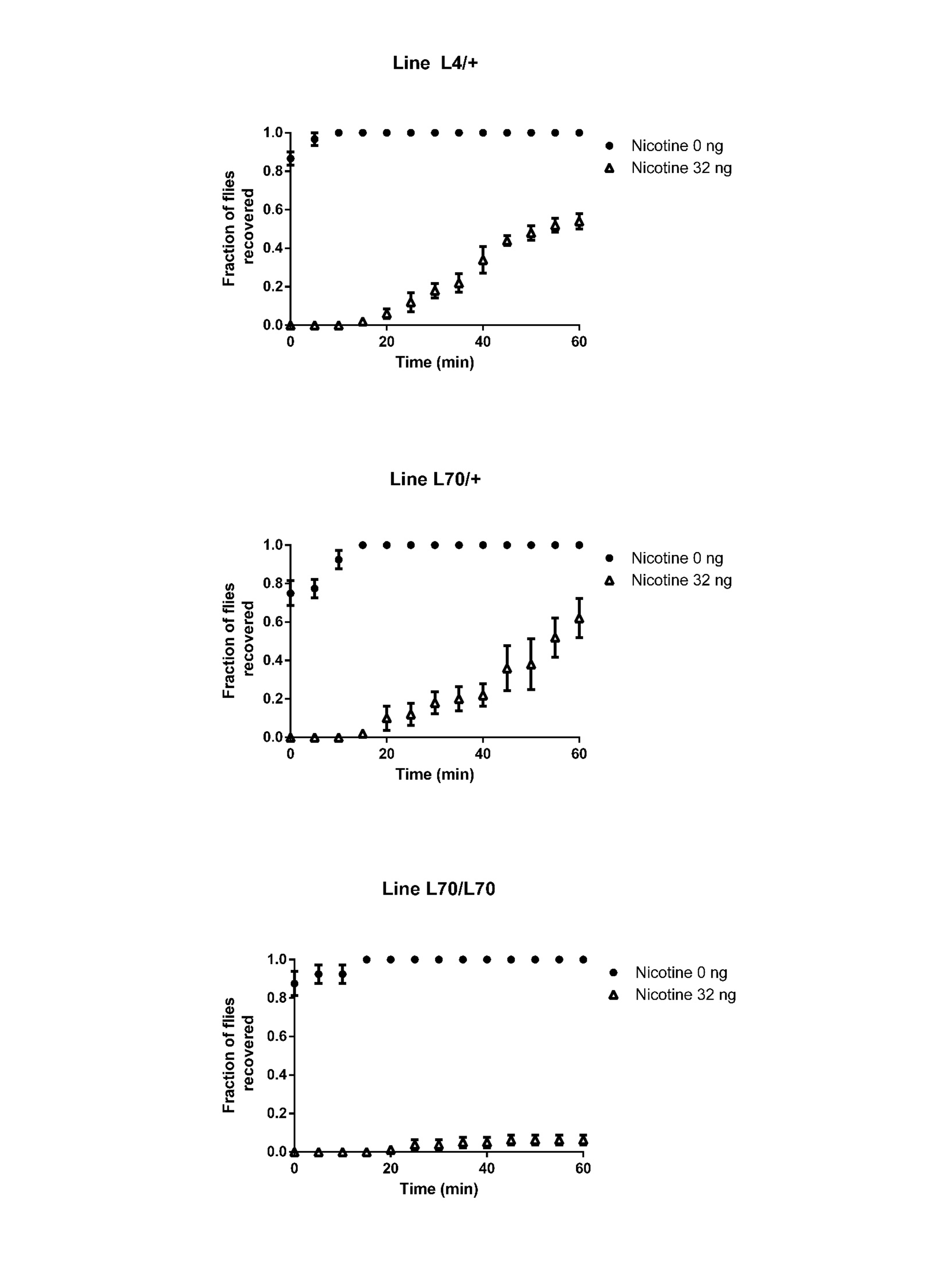

Supplement: S1 Fig — All mutant genotypes recovered immediately after exposure to vaporized water (nicotine 0 ng). (TIF) [file pone.0133956.s001.tif]

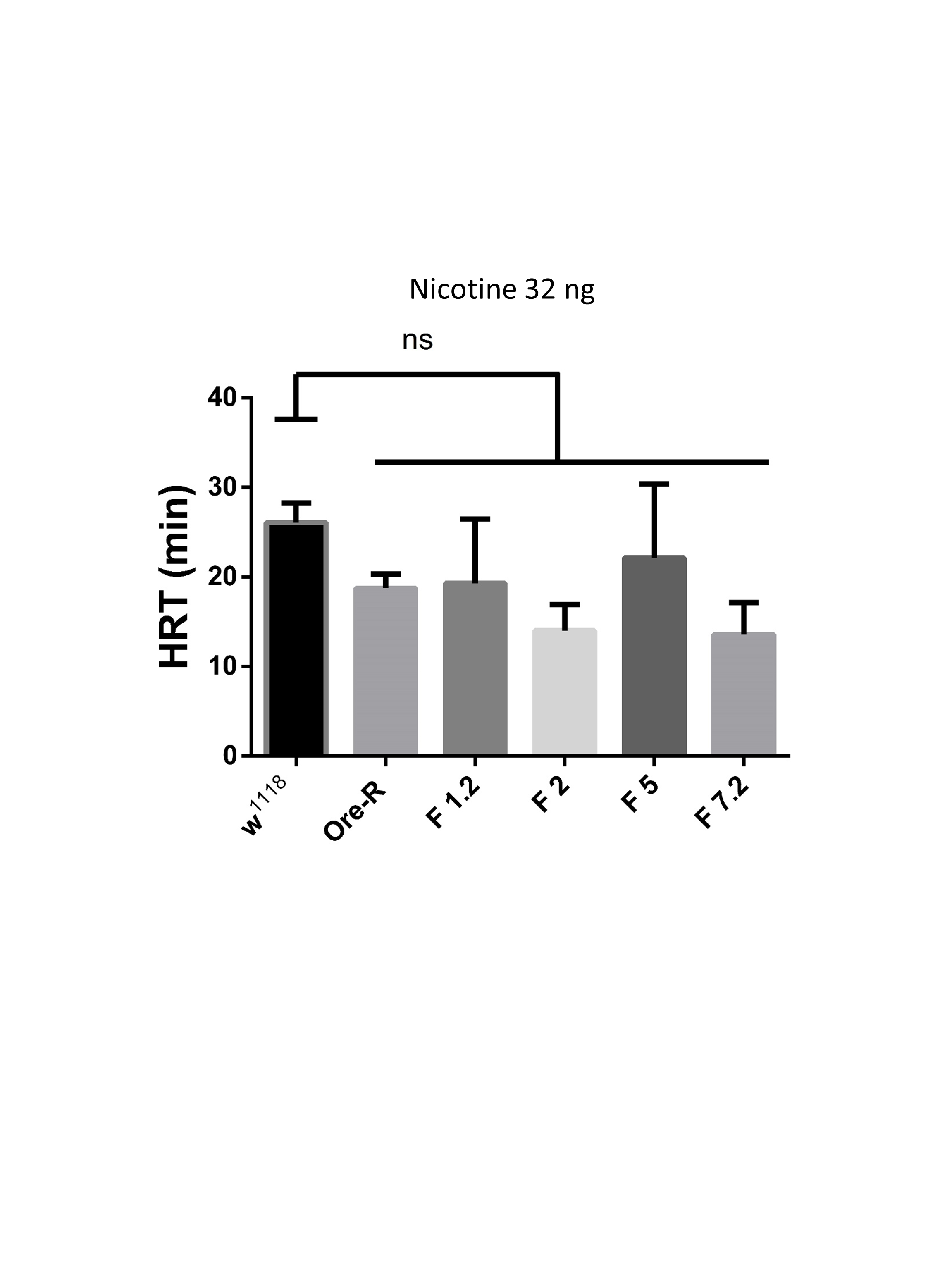

Supplement: S2 Fig — P{GawB} was mobilized using the transposase source Δ2–3. Five independent lines: F1.1, F1.2, F2, F5, F7.2 (data for F1.1 is in Fig 1) were recovered, all of them having a precise excision that was confirmed by DNA sequence. HRT from all revertant lines did not show significant differences from the control lines. ns = not significant. (TIF) [file pone.0133956.s002.tif]

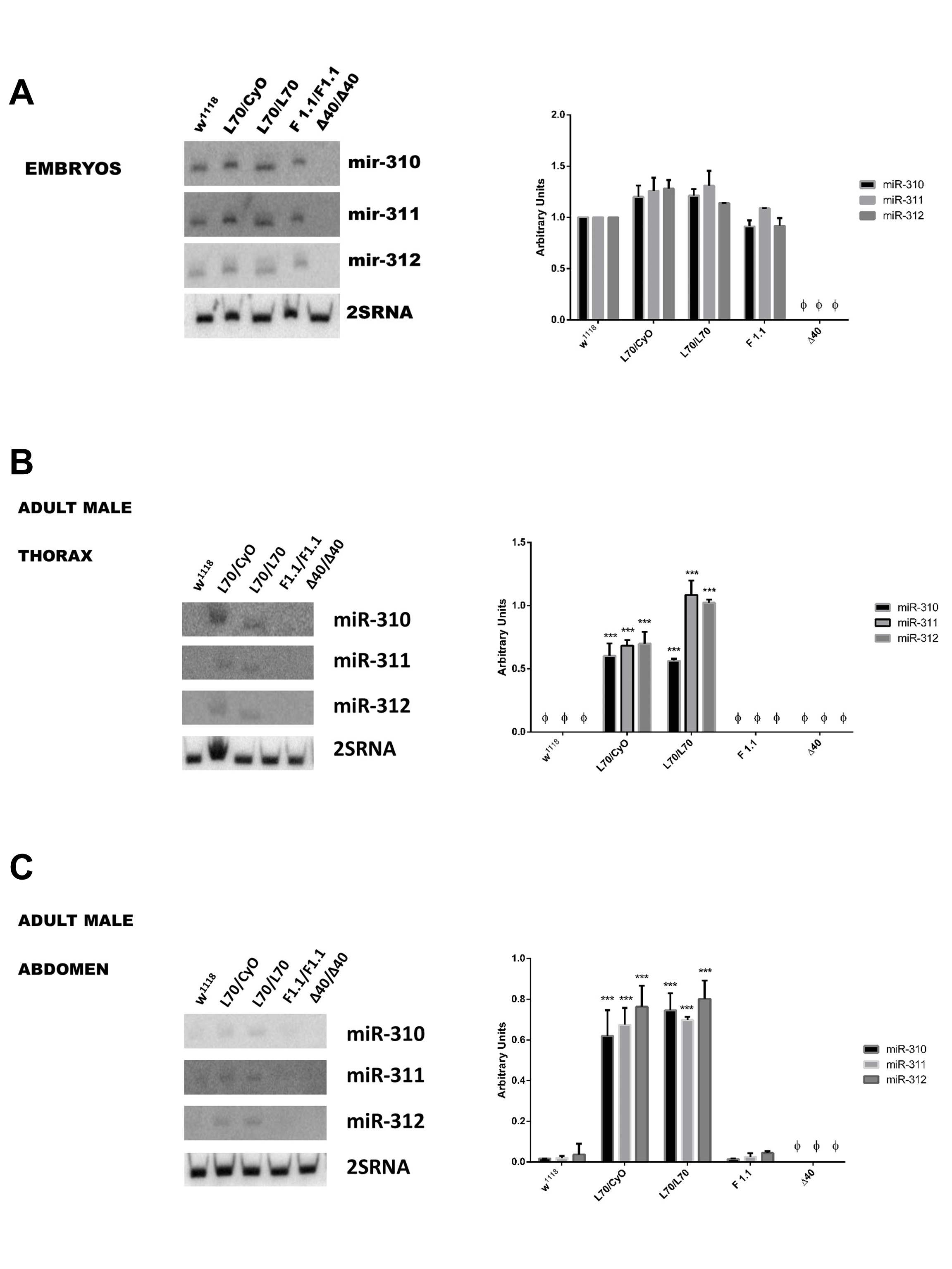

Supplement: S3 Fig — (A) Northern blot and their quantitative analysis using RNA from embryos. (B) Northern blot and their quantitative analysis using RNA from adult male thorax. (C) Northern blot and their quantitative analysis using RNA from adult male abdomen. Probes used are indicated at the right of each hybridization result. There is practically no adult expression of the miR-310, miR-311 and miR-312 transcripts from wt, Δ40 and F1.1 revertant line in adult tissues tested, while there is high expression of the transcripts in L70 mutant line. miR-313 was not detectable. *** = P << 0.001, Ф = non detectable. (TIF) [file pone.0133956.s003.tif]

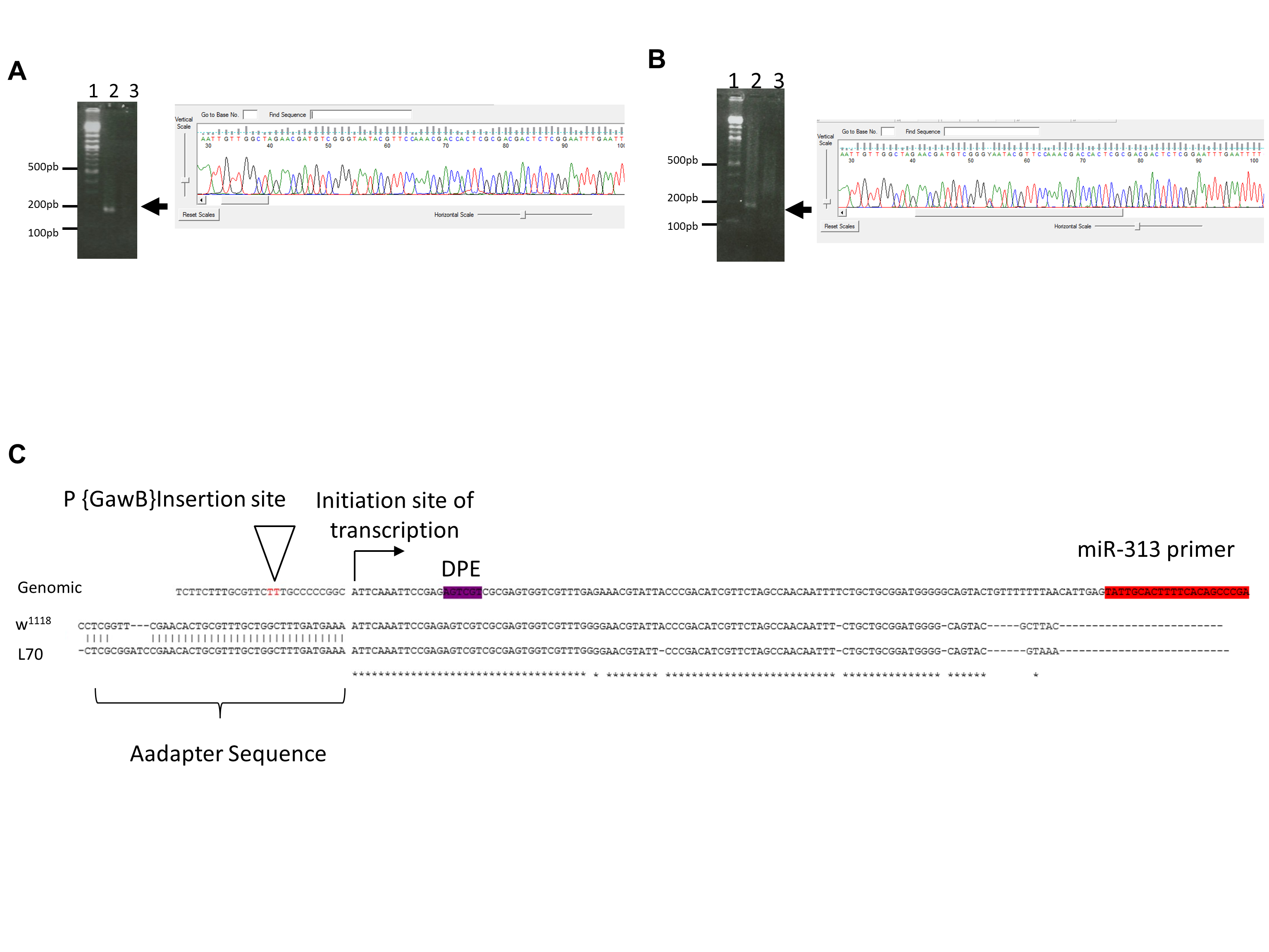

Supplement: S4 Fig — FirstChoice RLM-RACE Kit was used to determine the transcription initiation site of the miR-310c. A) Arrow shows third nested PCR product of w 1118 and sequence histogram. 1) MWM 2) Primers miR-313/Inner5´RACE, w 1118 cDNA. 3) Primers miR-313/Inner5´RACE, no cDNA B) Arrow shows third nested PCR product of L70 and sequence histogram. 1) MWM 2) Primers miR-313/Inner5´RACE, L70 cDNA. 3) Primers miR-313/Inner5´RACE, no cDNA C) Sequence alignment of w1118 and L70 PCR products. (TIF) [file pone.0133956.s004.tif]
