# Supplementary material for: The Esg Gene Is Involved in Nicotine Sensitivity in Drosophila melanogaster
Source: PLoS One. 2015 Jul 29;10(7):e0133956. doi: 10.1371/journal.pone.0133956 (PMC4519288; doi:10.1371/journal.pone.0133956)
Supplement: S5 Fig — (PDF) [file pone.0133956.s005.pdf]

EZBiolab
1033 3rd Avenue SW, Suite 215, Carmel, IN 46032 USA
www.ezbiolab.com
service@ezbiolab.com

Custom Antibody Product Report

Order Number: 082813AB0401

P.O. Number: CVMT13-6045

Customer Information

PATRICIA MORANWHITE
ConsuLAB-BQ SOS
451 Tecate Road, Suite #15,
Tecate, CA 91980
Phone: +(619) 478 5577
Fax: +(619) 566 2222
Email: info@consulab-bqsos.com
vnarvaezgm@gmail.com

Number of antibodies included in this package: 1
Number of antibodies have not been delivered: 0

All reagents are shipped as lyophilized powder. For research use only.

All claim should be made within 30 days of the products received at the destination in writing form (fax or email) and failure to do so shall constitute a waiver by customer for any and all such claims.

Antibody: gi|17136258|ref|NP_476600.1|escargot [Drosophilamelanogaster]
Antigen Sequence:

1. YQFEAPQNHSNTP-C
2. DEHIEEEDEDVDV-C
3. C-PNASAKKDKNQPP

Delivery List:

Antibody	List of tubes	Description	Rabbit #	Original serum vol.	Titer (ELISA)	Purification	IgG (mg/ml)
1		peptide, 2.3 mg, 74.25% YQFEAPQNHSNTP-C					
	1	Pre-immune sera	1	2 ml			
	2	Anti-serum		25 ml	>1:384,000	SAS	2mg/ml
	3	Anti-serum		25 ml	>1:384,000	SAS	2mg/ml
	4	Pre-immune sera	2	2 ml			
	5	Anti-serum		25 ml	>1:384,000	SAS	2mg/ml
	6	Anti-serum		25 ml	>1:384,000	SAS	2mg/ml
2		peptide, 2.3 mg, 74.18% DEHIEEEDEDVDV-C					
	1	Pre-immune sera	1	2 ml			
	2	Anti-serum		25 ml	>1:384,000	SAS	2mg/ml
	3	Anti-serum		25 ml	>1:384,000	SAS	2mg/ml
	4	Pre-immune sera	2	2 ml			
	5	Anti-serum		25 ml	>1:384,000	SAS	2mg/ml
	6	Anti-serum		25 ml	>1:384,000	SAS	2mg/ml
3		peptide, 2.3 mg, 93.26% C-PNASAKKDKNQPP					
	1	Pre-immune sera	1	2 ml			
	2	Anti-serum		25 ml	>1:384,000	SAS	2mg/ml
	3	Anti-serum		25 ml	>1:384,000	SAS	2mg/ml
	4	Pre-immune sera	2	2 ml			
	5	Anti-serum		25 ml	>1:384,000	SAS	2mg/ml
	6	Anti-serum		25 ml	>1:384,000	SAS	2mg/ml

Formulation: Lyophilized powder.

SAS Purification Procedure

1 Ammonium sulfate precipitation (50% saturation)
2 Dialysis dialysis buffer: 0.5 x PBS (pH 7.4)
3 freeze drying
4 Add Pure H_2O to the original volume to dissolve the powders.

Determination of antibody concentration: BCA protein assays (PIERCE 23227)

Storage: Antibody stock solutions (e.g., 1-5 mg/ml) often may be stored at 4 ℃ for days to weeks without significant loss in activity. For increased stability, glycerol or ethylene glycol may be added to a final concentration of 50% and the antibody stored at -20 ℃. Alternatively, the antibody solution may be stored in small working aliquots at -20 ℃ to avoid repeated freeze-thaw cycles.

<u>Reconstitution of Sample</u>

Marker：Pierce, PageRuler Prestained Protein Ladder

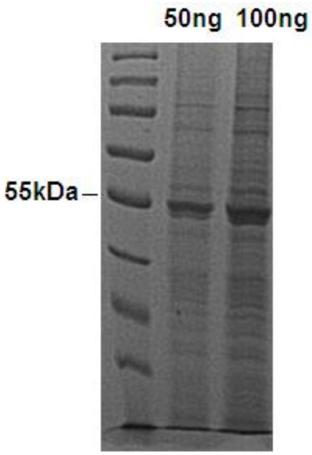

Result of Western Blot

Pre-immune sera

Diluent fold of primary antibody: **1:2000**
Diluent fold of secondary anitbody: **1:5000**

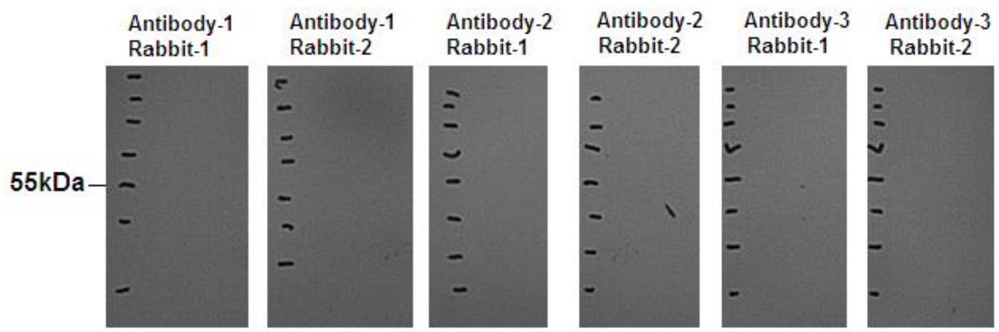

Anti-serum

Diluent fold of primary antibody: **1:2000**
Diluent fold of secondary anitbody: **1:5000**

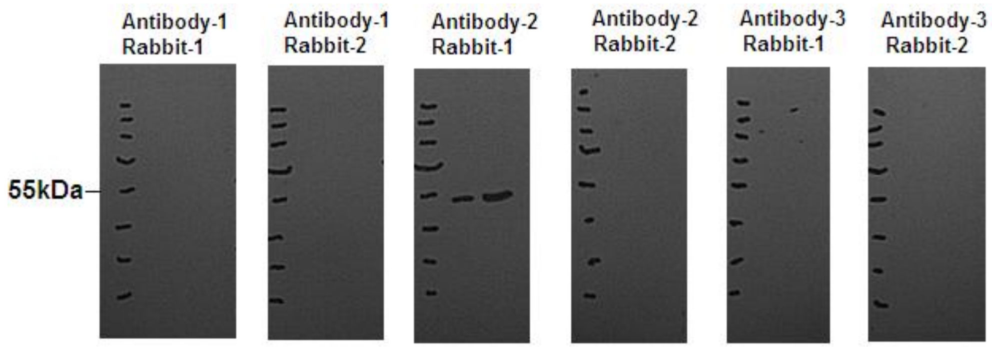

Diluent fold of primary antibody: **1:1000**
Diluent fold of secondary anitbody: **1:5000**

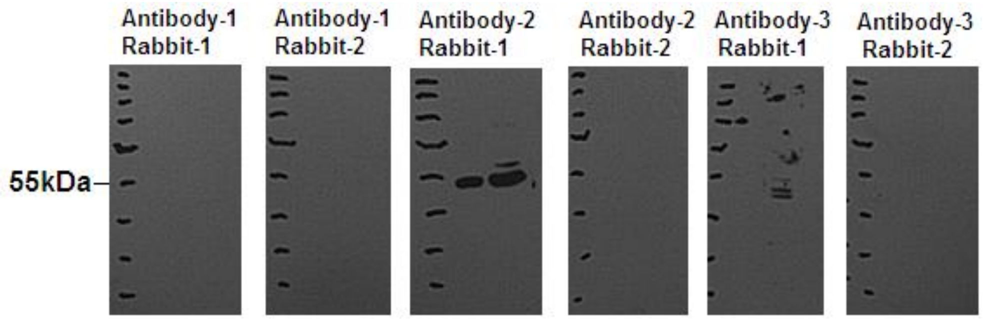

Recommend use of antibody：**1:2000**
