# Supplementary material for: The Esg Gene Is Involved in Nicotine Sensitivity in Drosophila melanogaster
Source: PLoS One. 2015 Jul 29;10(7):e0133956. doi: 10.1371/journal.pone.0133956 (PMC4519288; doi:10.1371/journal.pone.0133956)
Supplement: S1 Table — (DOCX) [file pone.0133956.s006.docx]

**Table S1. Drivers used for the expression of the *miR-310^c^*.**

| **Stock** | **Genotype** | **% Viability** | **Expression pattern** | **HRT (min)** |
| --- | --- | --- | --- | --- |
| 25374 | y^1^ w*; P{Act5C-GAL4-w}E1/CyO | 0 | Strong constitutive | --- |
| 108069 | y^1^w^*^;P{tubP-GAL4}LL7/TM3,Sb^1^ | 0 | Strong constitutive | --- |
| 3041 | y^1^w^1118^;P{GawB}ap^md544^/CyO | 0 | early development | --- |
| 7415 | w^1118^;P{GAL4}repo/TM3,Sb^1^ | 0 | glial | --- |
| 5398 | w^*^;P{GawB}332.3 | 0 | amnioserosa | --- |
| 8860 | w^1118^P{GawB}Bx^MS1096^ | 5 | wing | ** |
| 113798 | y^*^w^*^;P{GawB}NP5941/CyO,P{UAS-lacZ.UW14}UW14 | 100 | miR-310*^c^* | 16±6 |
| 8760 | w*; P{GAL4-elav.L}3 | 100 | pan neural | 20±8 |
| 6870 | w^1118^;P{Sgs3-GAL4.PD}TP1 | 100 | salivary glands | 26±5 |
| 1560 | w^*^;P{GAL4-arm.S}11 | 100 | weak constitutive | 22±10 |
| 1774 | w^*^;P{GawB}69B | 100 | ectoderm | 28±8 |
| 38464 | w^*^;P{Mhc-RFP.F3-580}2,P{Mhc-GAL4.F3-580}2/SM6b | 100 | muscle | 22±8 |
| 26875 | y^1^w^*^;P{nullo-GAL4.G}5.20 | 100 | Weak constitutive | 17±7 |
| 9146 | w^1118^;P{GMR-GAL4.w^-^}2/CyO | 100 | eye | 26±14 |

Fourteen different drivers were used to try to phenocopy L70. Out of this collection, five were lethal and one was semi-lethal. The rest were viable but did not phenocopy L70. n≥ 200 individuals. ^**^MS1096 had wing defects, locomotion deficiencies and died within the first two days after eclosion.
