# Supplementary material for: The Esg Gene Is Involved in Nicotine Sensitivity in Drosophila melanogaster
Source: PLoS One. 2015 Jul 29;10(7):e0133956. doi: 10.1371/journal.pone.0133956 (PMC4519288; doi:10.1371/journal.pone.0133956)
Supplement: S2 Table — (DOCX) [file pone.0133956.s007.docx]

| Analyzed List: | **L4 mean log2ratio > 1** | | | | | |
| --- | --- | --- | --- | --- | --- | --- |
| Analysis Type: | PANTHER Overrepresentation Test (release 20150430) | | | | | |
| Annotation Version and Release Date: | GO Ontology database Released 2015-05-09 | | | | | |
| Reference List: | Drosophila melanogaster (all genes in database) | | | | | |
| Bonferroni correction: | true |  |  |  |  |  |
| GO biological process experimental only | Drosophila melanogaster - REFLIST (13690) | Number of genes (507) | expected | over/under | fold Enrichment | P-value |
| spermatogenesis, exchange of chromosomal proteins | 4 | 4 | 0.15 | + | > 5 | 3.65E-02 |
| sperm chromatin condensation | 4 | 4 | 0.15 | + | > 5 | 3.65E-02 |
| spermatid differentiation | 114 | 29 | 4.22 | + | > 5 | 3.03E-12 |
| spermatid development | 99 | 25 | 3.67 | + | > 5 | 3.26E-10 |
| male gamete generation | 217 | 51 | 8.04 | + | > 5 | 1.14E-21 |
| spermatogenesis | 215 | 49 | 7.96 | + | > 5 | 3.65E-20 |
| detection of chemical stimulus involved in sensory perception | 66 | 12 | 2.44 | + | 4.91 | 1.93E-02 |
| male meiosis | 73 | 13 | 2.7 | + | 4.81 | 1.03E-02 |
| detection of stimulus involved in sensory perception | 87 | 15 | 3.22 | + | 4.66 | 2.90E-03 |
| sensory perception of chemical stimulus | 114 | 16 | 4.22 | + | 3.79 | 1.72E-02 |
| meiotic cell cycle | 168 | 23 | 6.22 | + | 3.7 | 2.96E-04 |
| meiotic nuclear division | 162 | 22 | 6 | + | 3.67 | 6.32E-04 |
| meiotic cell cycle process | 133 | 17 | 4.93 | + | 3.45 | 2.98E-02 |
| multicellular organism reproduction | 1056 | 125 | 39.11 | + | 3.2 | 4.20E-28 |
| reproduction | 1177 | 138 | 43.59 | + | 3.17 | 3.02E-31 |
| sensory perception | 196 | 21 | 7.26 | + | 2.89 | 4.21E-02 |
| nuclear division | 315 | 32 | 11.67 | + | 2.74 | 9.50E-04 |
| organelle fission | 318 | 32 | 11.78 | + | 2.72 | 1.16E-03 |
| sexual reproduction | 876 | 87 | 32.44 | + | 2.68 | 1.46E-13 |
| multi-organism reproductive process | 883 | 87 | 32.7 | + | 2.66 | 2.32E-13 |
| multicellular organismal reproductive process | 853 | 84 | 31.59 | + | 2.66 | 8.87E-13 |
| gamete generation | 756 | 74 | 28 | + | 2.64 | 8.80E-11 |
| reproductive process | 941 | 90 | 34.85 | + | 2.58 | 3.72E-13 |
| single organism reproductive process | 816 | 76 | 30.22 | + | 2.51 | 4.53E-10 |
| multi-organism process | 1138 | 101 | 42.15 | + | 2.4 | 4.88E-13 |
| developmental process involved in reproduction | 729 | 60 | 27 | + | 2.22 | 2.00E-05 |
| cellular process involved in reproduction in multicellular organism | 701 | 57 | 25.96 | + | 2.2 | 7.30E-05 |
| germ cell development | 633 | 48 | 23.44 | + | 2.05 | 6.38E-03 |
| multicellular organismal process | 3307 | 188 | 122.47 | + | 1.54 | 1.27E-07 |
| biological_process | 5288 | 257 | 195.84 | + | 1.31 | 4.93E-05 |
| Unclassified | 8402 | 252 | 311.16 | - | 0.81 | 0.00E+00 |
| wing disc development | 455 | 2 | 16.85 | - | < 0.2 | 1.27E-02 |

| **Genes upregulated in L4 involved in Sensory Perception** | | | |
| --- | --- | --- | --- |
| Gr64a | Gustatory receptor for sugar taste 64a;Gr64a;ortholog | GUSTATORY RECEPTOR FOR SUGAR TASTE 61A-RELATED (PTHR21421:SF26) |  |
| unc | Uncoordinated;unc;ortholog |  |  |
| Or85b | Odorant receptor 85b;Or85b;ortholog | ODORANT RECEPTOR 13A-RELATED (PTHR21137:SF7) | G-protein coupled receptor |
| Or22b | Odorant receptor 22b;Or22b;ortholog | ODORANT RECEPTOR 22A-RELATED (PTHR21137:SF28) | G-protein coupled receptor |
| Or88a | Odorant receptor 88a;Or88a;ortholog | ODORANT RECEPTOR 13A-RELATED (PTHR21137:SF7) | G-protein coupled receptor |
| brv2 | AT14535p;brv2;ortholog |  |  |
| ppk11 | Pickpocket protein 11;ppk11;ortholog | PICKPOCKET 16-RELATED (PTHR11690:SF150) | ion channel |
| Ir75a | Ionotropic receptor 75a;Ir75a;ortholog | IONOTROPIC RECEPTOR 75A (PTHR18966:SF187) | ionotropic glutamate receptor;ionotropic glutamate receptor |
| brv1 | Brivido-1;brv1;ortholog | BRIVIDO-1-RELATED (PTHR10877:SF138) | ion channel;membrane-bound signaling molecule;G-protein modulator |
| Gr59c | Putative gustatory receptor 59c;Gr59c;ortholog | GUSTATORY AND PHEROMONE RECEPTOR 33A-RELATED (PTHR21143:SF16) | G-protein coupled receptor |
| Dhc36C | Dynein heavy chain at 36C;Dhc36C;ortholog | DYNEIN HEAVY CHAIN AT 36C (PTHR10676:SF281) | hydrolase;microtubule binding motor protein |
| Ir7a | Ionotropic receptor 7a;Ir7a;ortholog | IONOTROPIC RECEPTOR 67C-RELATED (PTHR18966:SF188) |  |
| Or74a | Odorant receptor 74a;Or74a;ortholog | ODORANT RECEPTOR 35A-RELATED (PTHR21137:SF2) | G-protein coupled receptor |
| Or82a | Odorant receptor 82a;Or82a;ortholog | ODORANT RECEPTOR 45A-RELATED (PTHR21137:SF15) | G-protein coupled receptor |
| rpk | Amiloride-sensitive Na+ channel;rpk;ortholog | AMILORIDE-SENSITIVE NA+ CHANNEL (PTHR11690:SF166) | ion channel |
| Ir75b | Ir75b;Ir75b;ortholog | IR75B-RELATED (PTHR18966:SF193) | ionotropic glutamate receptor;ionotropic glutamate receptor |
| Or30a | Odorant receptor 30a;Or30a;ortholog | ODORANT RECEPTOR 30A-RELATED (PTHR21137:SF3) | G-protein coupled receptor |
| Ir40a | Ionotropic receptor 40a, isoform F;Ir40a;ortholog | IONOTROPIC RECEPTOR 40A, ISOFORM F-RELATED (PTHR18966:SF2) |  |
| Or67c | Odorant receptor 67c;Or67c;ortholog | ODORANT RECEPTOR 13A-RELATED (PTHR21137:SF7) | G-protein coupled receptor |
| Or45a | Odorant receptor 45a;Or45a;ortholog | ODORANT RECEPTOR 45A-RELATED (PTHR21137:SF15) | G-protein coupled receptor |
| Or59b | Odorant receptor 59b;Or59b;ortholog | ODORANT RECEPTOR 13A-RELATED (PTHR21137:SF7) | G-protein coupled receptor |

| Analyzed List: | **L4 mean log2raio < -1** | | | | | |
| --- | --- | --- | --- | --- | --- | --- |
| Analysis Type: | PANTHER Overrepresentation Test (release 20150430) | | | | | |
| Annotation Version and Release Date: | GO Ontology database Released 2015-05-09 | | | | | |
| Reference List: | Drosophila melanogaster (all genes in database) | | | | | |
| Bonferroni correction: | true |  |  |  |  |  |
| GO biological process experimental only | Drosophila melanogaster - REFLIST (13690) | Number of genes (233) | expected | over/under | fold Enrichment | P-value |
| detection of pheromone | 14 | 5 | 0.24 | + | > 5 | 1.05E-02 |
| response to pheromone | 19 | 6 | 0.32 | + | > 5 | 2.36E-03 |
| cuticle development | 82 | 9 | 1.4 | + | > 5 | 2.95E-02 |
| Unclassified | 8402 | 116 | 143 | - | 0.81 | 0.00E+00 |

| **Genes downregulated in L4 involved in response to pheromone** | | |
| --- | --- | --- |
| CheB53a | Chemosensory protein B 53a;CheB53a;ortholog |  |
| CheB98a | Chemosensory protein A 98a;CheB98a;ortholog |  |
| lush | General odorant-binding protein lush;lush;ortholog | GENERAL ODORANT-BINDING PROTEIN LUSH (PTHR21364:SF1) |
| CheB38a | Chemosensory protein B 38a;CheB38a;ortholog |  |
| CheB53b | Chemosensory protein B 53b;CheB53b;ortholog |  |
| CheB74a | Chemosensory protein B 74a;CheB74a;ortholog |  |

| **Genes downregulated in L4 involved in cuticle development** | | | |
| --- | --- | --- | --- |
| burs | Bursicon;Burs;ortholog |  |  |
| TwdlF | LD19715p;TwdlF;ortholog | FI07246P-RELATED (PTHR31927:SF2) |  |
| kkv | Krotzkopf verkehrt, isoform A;kkv;ortholog | KROTZKOPF VERKEHRT, ISOFORM A (PTHR22914:SF8) | transferase |
| knk | Knickkopf;knk;ortholog | KNICKKOPF (PTHR24036:SF8) |  |
| y | Protein yellow;y;ortholog | PROTEIN YELLOW (PTHR10009:SF14) |  |
| dsx-c73A | Doublesex cognate 73A;dsx-c73A;ortholog |  |  |
| m | Miniature;m;ortholog | MINIATURE (PTHR22907:SF5) |  |
| laccase2 | CG42345, isoform E;laccase2;ortholog | LACCASE-6 (PTHR11709:SF33) | oxidase |
| obst-A | LD43683p;obst-A;ortholog | LD43683P (PTHR23301:SF9) |  |
