# Supplementary material for: The Esg Gene Is Involved in Nicotine Sensitivity in Drosophila melanogaster
Source: PLoS One. 2015 Jul 29;10(7):e0133956. doi: 10.1371/journal.pone.0133956 (PMC4519288; doi:10.1371/journal.pone.0133956)
Supplement: S3 Table — (DOCX) [file pone.0133956.s008.docx]

| Analyzed List: | **L70 mean log2ratio > 1** | | | | | |
| --- | --- | --- | --- | --- | --- | --- |
| Analysis Type: | PANTHER Overrepresentation Test (release 20150430) | | | | | |
| Annotation Version and Release Date: | GO Ontology database Released 2015-05-09 | | | | | |
| Reference List: | Drosophila melanogaster (all genes in database) | | | | | |
| Bonferroni correction: | true | | | | | |
| GO biological process experimental only | Drosophila melanogaster - REFLIST (13690) | Number of genes (164) | expected | over/under | fold Enrichment | P-value |
| detection of chemical stimulus involved in sensory perception of smell | 53 | 8 | 0.63 | + | > 5 | 6.68E-04 |
| detection of chemical stimulus involved in sensory perception | 66 | 8 | 0.79 | + | > 5 | 3.39E-03 |
| detection of stimulus involved in sensory perception | 87 | 9 | 1.04 | + | > 5 | 2.73E-03 |
| sensory perception of smell | 81 | 8 | 0.97 | + | > 5 | 1.50E-02 |
| detection of chemical stimulus | 83 | 8 | 0.99 | + | > 5 | 1.78E-02 |
| sensory perception of chemical stimulus | 114 | 10 | 1.37 | + | > 5 | 3.04E-03 |
| sensory perception | 196 | 14 | 2.35 | + | > 5 | 2.79E-04 |
| detection of stimulus | 144 | 10 | 1.73 | + | > 5 | 2.32E-02 |
| neurological system process | 347 | 15 | 4.16 | + | 3.61 | 4.48E-02 |
| system process | 392 | 16 | 4.7 | + | 3.41 | 4.72E-02 |
| Unclassified | 8402 | 77 | 100.65 | - | 0.77 | 0.00E+00 |

| **Genes upregulated in L70 involved in sensory perception** | | | |
| --- | --- | --- | --- |
| unc | Uncoordinated;unc;ortholog |  |  |
| Or85b | Odorant receptor 85b;Or85b;ortholog | ODORANT RECEPTOR 13A-RELATED (PTHR21137:SF7) | G-protein coupled receptor |
| Or88a | Odorant receptor 88a;Or88a;ortholog | ODORANT RECEPTOR 13A-RELATED (PTHR21137:SF7) | G-protein coupled receptor |
| brv2 | AT14535p;brv2;ortholog |  |  |
| Or22a | Odorant receptor 22a;Or22a;ortholog | ODORANT RECEPTOR 22A-RELATED (PTHR21137:SF28) | G-protein coupled receptor |
| ppk11 | Pickpocket protein 11;ppk11;ortholog | PICKPOCKET 16-RELATED (PTHR11690:SF150) | ion channel |
| Ir84a | Ionotropic receptor 84a;Ir84a;ortholog | IONOTROPIC RECEPTOR 67C-RELATED (PTHR18966:SF188) |  |
| Or33b | Odorant receptor 33b;Or33b;ortholog | ODORANT RECEPTOR 13A-RELATED (PTHR21137:SF7) | G-protein coupled receptor |
| Dhc93AB | Dynein heavy chain at 93AB;Dhc93AB;ortholog | DYNEIN HEAVY CHAIN AT 93AB (PTHR10676:SF276) | hydrolase;microtubule binding motor protein |
| ato | Protein atonal;ato;ortholog | PROTEIN ATONAL (PTHR19290:SF84) | basic helix-loop-helix transcription factor;nuclease |
| Gr64d | Gustatory receptor for sugar taste 64d;Gr64d;ortholog | GUSTATORY RECEPTOR FOR SUGAR TASTE 64C-RELATED (PTHR21421:SF28) |  |
| Or9a | Odorant receptor 9a;Or9a;ortholog | ODORANT RECEPTOR 47A-RELATED (PTHR21137:SF26) | G-protein coupled receptor |
| Ir40a | Ionotropic receptor 40a, isoform F;Ir40a;ortholog | IONOTROPIC RECEPTOR 40A, ISOFORM F-RELATED (PTHR18966:SF2) |  |
| Or45a | Odorant receptor 45a;Or45a;ortholog | ODORANT RECEPTOR 45A-RELATED (PTHR21137:SF15) | G-protein coupled receptor |

| Analyzed List: | **L70 mean log2ratio < -1** | | | | | |
| --- | --- | --- | --- | --- | --- | --- |
| Analysis Type: | PANTHER Overrepresentation Test (release 20150430) | | | | | |
| Annotation Version and Release Date: | GO Ontology database Released 2015-05-09 | | | | | |
| Reference List: | Drosophila melanogaster (all genes in database) | | | | | |
| Bonferroni correction: | true | | | | | |
| GO biological process experimental only | Drosophila melanogaster - REFLIST (13690) | Number of genes (445) | expected | over/under | fold Enrichment | P-value |
| detection of pheromone | 14 | 9 | 0.46 | + | > 5 | 2.95E-06 |
| response to pheromone | 19 | 10 | 0.62 | + | > 5 | 2.41E-06 |
| detection of chemical stimulus | 83 | 21 | 2.7 | + | > 5 | 2.43E-09 |
| detection of chemical stimulus involved in sensory perception of smell | 53 | 10 | 1.72 | + | > 5 | 2.59E-02 |
| detection of chemical stimulus involved in sensory perception | 66 | 12 | 2.15 | + | > 5 | 5.20E-03 |
| sensory perception of smell | 81 | 14 | 2.63 | + | > 5 | 1.39E-03 |
| detection of stimulus | 144 | 22 | 4.68 | + | 4.7 | 8.60E-06 |
| detection of stimulus involved in sensory perception | 87 | 13 | 2.83 | + | 4.6 | 1.63E-02 |
| sensory perception of chemical stimulus | 114 | 17 | 3.71 | + | 4.59 | 6.96E-04 |
| reproduction | 1177 | 94 | 38.26 | + | 2.46 | 8.64E-13 |
| multicellular organism reproduction | 1056 | 76 | 34.33 | + | 2.21 | 1.41E-07 |
| sexual reproduction | 876 | 54 | 28.47 | + | 1.9 | 1.23E-02 |
| multi-organism reproductive process | 883 | 54 | 28.7 | + | 1.88 | 1.54E-02 |
| reproductive process | 941 | 57 | 30.59 | + | 1.86 | 1.11E-02 |
| multi-organism process | 1138 | 66 | 36.99 | + | 1.78 | 7.78E-03 |
| biological_process | 5288 | 224 | 171.89 | + | 1.3 | 7.30E-04 |
| Unclassified | 8402 | 222 | 273.11 | - | 0.81 | 0.00E+00 |
| system development | 2141 | 37 | 69.59 | - | 0.53 | 6.76E-03 |
| neurogenesis | 1352 | 18 | 43.95 | - | 0.41 | 7.09E-03 |
| nervous system development | 1482 | 19 | 48.17 | - | 0.39 | 1.07E-03 |
| generation of neurons | 835 | 8 | 27.14 | - | 0.29 | 2.21E-02 |
| transport | 657 | 5 | 21.36 | - | 0.23 | 3.81E-02 |
| neuron development | 631 | 4 | 20.51 | - | < 0.2 | 1.68E-02 |

| **Genes downregulated in L70 involved in detection of chemical stimulus** | | | |
| --- | --- | --- | --- |
| Or42a | Odorant receptor 42a;Or42a;ortholog | ODORANT RECEPTOR 13A-RELATED (PTHR21137:SF7) | G-protein coupled receptor |
| CheB42c | Chemosensory protein B 42c;CheB42c;ortholog |  |  |
| Or49a | Odorant receptor 49a;Or49a;ortholog | ODORANT RECEPTOR 13A-RELATED (PTHR21137:SF7) | G-protein coupled receptor |
| CheB38b | Chemosensory protein B 38b;CheB38b;ortholog |  |  |
| CheB42b | Chemosensory protein B 42b;CheB42b;ortholog |  |  |
| CheB98a | Chemosensory protein A 98a;CheB98a;ortholog |  |  |
| Or94a | Odorant receptor 94a;Or94a;ortholog | ODORANT RECEPTOR 46A, ISOFORM B-RELATED (PTHR21137:SF23) | G-protein coupled receptor |
| Gr63a | Gustatory and odorant receptor 63a;Gr63a;ortholog | GUSTATORY AND ODORANT RECEPTOR 63A (PTHR21143:SF84) | G-protein coupled receptor |
| Ir75c | Ir75c;Ir75c;ortholog | IR75B-RELATED (PTHR18966:SF193) | ionotropic glutamate receptor;ionotropic glutamate receptor |
| Or67a | Odorant receptor 67a;Or67a;ortholog | ODORANT RECEPTOR 13A-RELATED (PTHR21137:SF7) | G-protein coupled receptor |
| Ir21a | Ionotropic receptor 21a;Ir21a;ortholog | IONOTROPIC RECEPTOR 21A (PTHR18966:SF236) | ionotropic glutamate receptor;ionotropic glutamate receptor |
| Or46a | Odorant receptor 46a, isoform B;Or46a;ortholog | ODORANT RECEPTOR 46A, ISOFORM B-RELATED (PTHR21137:SF23) | G-protein coupled receptor |
| CheB38a | Chemosensory protein B 38a;CheB38a;ortholog |  |  |
| Or67d | Odorant receptor 67d;Or67d;ortholog | ODORANT RECEPTOR 35A-RELATED (PTHR21137:SF2) | G-protein coupled receptor |
| Ir8a | Ionotropic receptor 8a;Ir8a;ortholog | IONOTROPIC RECEPTOR 8A (PTHR18966:SF212) | ionotropic glutamate receptor;ionotropic glutamate receptor |
| CheA29a | Chemosensory protein A 29a;CheA29a;ortholog | CHEMOSENSORY PROTEIN A 29A-RELATED (PTHR21112:SF0) |  |
| Ir76a | Ionotropic receptor 76a, isoform E;Ir76a;ortholog | IONOTROPIC RECEPTOR 41A-RELATED (PTHR18966:SF226) | ionotropic glutamate receptor;ionotropic glutamate receptor |
| CheB53b | Chemosensory protein B 53b;CheB53b;ortholog |  |  |
| CheB74a | Chemosensory protein B 74a;CheB74a;ortholog |  |  |
| Or83c | Putative odorant receptor 83c;Or83c;ortholog | ODORANT RECEPTOR 35A-RELATED (PTHR21137:SF2) | G-protein coupled receptor |
| Or59b | Odorant receptor 59b;Or59b;ortholog | ODORANT RECEPTOR 13A-RELATED (PTHR21137:SF7) | G-protein coupled receptor |
